# Non-autonomous regulation of neurogenesis by extrinsic cues: a *Drosophila* perspective

**DOI:** 10.1093/oons/kvac004

**Published:** 2022-05-04

**Authors:** Phuong-Khanh Nguyen, Louise Y Cheng

**Affiliations:** Peter MacCallum Cancer Centre, Melbourne, Victoria 3000, Australia; Department of Anatomy and Physiology, The University of Melbourne, Victoria 3010, Australia; Peter MacCallum Cancer Centre, Melbourne, Victoria 3000, Australia; Sir Peter MacCallum Department of Oncology, The University of Melbourne, Victoria 3010, Australia; Department of Anatomy and Physiology, The University of Melbourne, Victoria 3010, Australia

**Keywords:** neuroblast, *Drosophila*, niche

## Abstract

The formation of a functional circuitry in the central nervous system (CNS) requires the correct number and subtypes of neural cells. In the developing brain, neural stem cells (NSCs) self-renew while giving rise to progenitors that in turn generate differentiated progeny. As such, the size and the diversity of cells that make up the functional CNS depend on the proliferative properties of NSCs. In the fruit fly *Drosophila*, where the process of neurogenesis has been extensively investigated, extrinsic factors such as the microenvironment of NSCs, nutrients, oxygen levels and systemic signals have been identified as regulators of NSC proliferation. Here, we review decades of work that explores how extrinsic signals non-autonomously regulate key NSC characteristics such as quiescence, proliferation and termination in the fly.

## INTRODUCTION

The final size of organs is specified by both intrinsic and extrinsic mechanisms during development. The intrinsic program of cell growth, proliferation, differentiation and death is governed by spatiotemporal expression of tissue-intrinsic factors, cell–cell interactions as well as epigenetic regulators [1]. This program receives further inputs from tissue-extrinsic cues, such as nutrients, oxygen and humoral signalling molecules [2]. As such, the coordination between extrinsic regulatory signals and tissue-intrinsic machinery fine tunes tissue growth and maturation in order to generate organs that are appropriately scaled to overall body size.

The central nervous system (CNS) is the cognitive control centre of the body that is generated from a small population of neural stem-progenitor cells during mammalian development. Neural stem cells (NSCs) first undergo symmetric cell division to expand the stem cell pool while later switch to asymmetric cell division [3]. Thus, NSCs are capable of self-renewal and are also multipotent, capable of producing various types of neural cells including neural progenitors [3]. Neural progenitors then divide asymmetrically to self-renew and concomitantly generate a postmitotic neural cell, or symmetrically to give rise to two postmitotic cells [3]. The ultimate function of the adult CNS is dictated by neural cell number and cellular diversity, both governed by multiple parameters determined during developmental neurogenesis. These include the NSC lineage-specific mode of division, the length at which NSCs are engaged in the cell cycle, and the speed at which they proliferate.

**Figure 1:**
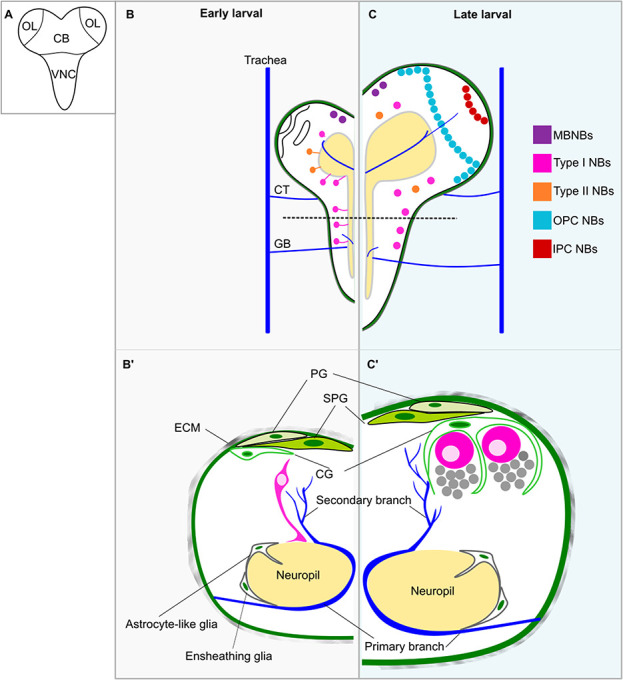
The cell types and their interactions within the developing CNS during early and late neural development. (**A)** The larval central nervous system (CNS) is divided into three neurogenic regions: the central brain (CB), the ventral nerve cord (VNC) and the optic lobe (OL). **(B–C′)** The neurogenic niche of neuroblasts (NBs) is composed of the extracellular matrix at the outer most layer of the CNS, different glial cell types (green) and the trachea (blue). The glial niche contains perineural glia (PG), subperineural glia (SPG), cortex glia (CG) and neuropil glia. Neuropil glia is consisted of ensheathing glia and astrocyte-like glia in which only astrocyte-like glia infiltrate the neuropil (yellow). The CNS primary trachea includes the cerebral trachea (CT) that invades the CB as well as the OL, and the ganglion branch (GB) that invades the VNC. Primary branches develop on the surface of the neuropil and are embedded between the neuropil and neuropil glia. From the primary branch, secondary tracheal branches elaborate towards the brain cortex. Dashed line indicates the cross-sections shown in B′ and C′. (B) Dorsal view of the CNS and (B′) cross-section of the VNC at the first-instar larval (L1) stage. At this stage, many NBs of type I (pink) and type II (orange) are in quiescence, each with a primary basal protrusion in contact with the neuropil. (C) Dorsal view of the CNS at the third-instar larval (L3) stage. At this stage, NBs of type I and type II are reactivated to resume postembryonic neurogenesis. In the OL, NBs in the OPC and the IPC are produced from two neuroepithelia. Note that mushroom body NBs (MBNBs, purple) do not become quiescent but continue proliferating throughout the embryonic-larval transition. (C′) Cross-section of the VNC at L3. During larval development, CG undergoes an extensive process of remodelling such that they extend their membrane to encase NBs and their progeny. As a result, CG forms chambers for each individual NB lineage. Concomitantly, the CNS trachea continues to elaborate towards the brain cortex.

In this review, we focus on studies conducted in the fruit fly *Drosophila melanogaster*, a model organism where NSC biology has been extensively studied*,* owing to its high genetic tractability. *Drosophila* NSCs, called neuroblasts, are specified from the neuroectoderm during embryonic development by the expression of proneural factors, Notch-mediated lateral inhibition and epithelial–mesenchymal transition (EMT) [4]. In the developing CNS of the fly, composed of three main neurogenic regions: the central brain (CB), the ventral nerve cord (VNC) and the optic lobe (OL), there are multiple types of neuroblasts that populate different sites, undergo distinct modes of proliferation and are engaged in the cell cycle for varied length of time (Fig. 1) [5, 6]. In the CB and the VNC, the majority are type I neuroblasts, which asymmetrically divide to self-renew and to generate a ganglion mother cell (GMC) that differentiates into two neurons or glial cells. Besides type I neuroblasts, in each brain hemisphere of the CB, there are eight type II neuroblasts that asymmetrically divide to self-renew and to generate an intermediate neural progenitor (INP) [7–10]. An INP then asymmetrically divides multiple times to produce GMCs. In addition, there are five mushroom body neuroblasts per brain hemisphere that undergo stereotypical type I division to generate neurons and glial cells of the mushroom body important for learning and memory of the animal [11, 12]. While neuroblasts in the CB and the VNC arise during embryogenesis, most neuroblasts of the OL are derived from the neuroepithelium during mid-larval development [13, 14]. Although OL neuroblasts also follow stereotypical type I mode of proliferation, they generate neurons and glial cells that make up the fly visual processing centre [15].

In the CNS, multiple intrinsic and extrinsic mechanisms coordinate together to regulate neuroblast behaviour, including quiescence, reactivation, proliferation and termination. As intrinsic regulators of neuroblast namely asymmetric division, cell fate determination and temporal patterning have been reviewed in detail elsewhere [4, 6, 16, 17], we will mostly focus our review on how extrinsic cues control the proliferative properties of neuroblasts.

## THE NEUROGENIC NICHE

The neurogenic microenvironment contains cellular and extracellular components that can regulate NSC behaviour through multiple mechanisms such as through the production of growth factors, morphogens and adhesion proteins [18]. In the developing CNS of *Drosophila*, the niche surrounding neuroblasts include the extracellular matrix (ECM), several types of neural cells (GMCs, neurons and glial cells) as well as the trachea. Together, these cell types can influence the proliferative properties of neuroblasts [19, 20] (Fig. 1).

### The ECM

The ECM resides at the outer surface of the fly CNS and is made up of various molecules, mainly including type IV Collagen (Col IV), Laminin, the heparan sulphate proteoglycan Perlecan and Nidogen [20–22] (Fig. 1). During embryogenesis, ECM components are deposited onto the embryonic brain by migratory macrophages to condense and to shape the CNS [23, 24]. As neurogenesis continues, the ECM needs to be remodelled to meet the increase in size of the CNS. To do so, surface glial cells (perineural and subperineural glia, see section 1.2) produce metalloprotease that can cleave ECM components such as those of the A disintegrin and metalloproteinase with thrombospondin motifs (AdamTS) family and Matrix metalloprotease 2 (Mmp2) [25, 26]. Although transcriptomic analysis showed that surface glia express high levels of *col IV*, whether glial Col IV functions in the preservation of the CNS structural integrity has not been examined [27]. After embryogenesis, the ECM of the CNS is largely produced by the fat body (equivalent to the mammalian liver) [21]. Since the knockdown of ECM components in the fat body could not fully deplete the ECM of the CNS, the ECM content of the CNS might not be exclusively produced by the fat body during larval stages [21]. Hence, it remains plausible that surface glia actively produce ECM components of the CNS after gliogenesis during embryonic development. Beside its structural roles, some studies have implicated ECM in the regulation of neuroblast behaviour such as reactivation and continued proliferation [28–32].

### The glial niche

The glial niche surrounding neuroblasts contains several types of glial cells that can be divided into three main groups based on their locations within the CNS: surface glia, cortex glia and neuropil glia [20, 33–35] (Fig. 1B′ and C′). Larval glial cells are mostly specified from neuroblasts during embryogenesis by the expression of the transcription factor (TF) Glial cell missing (Gcm), which acts as a binary switch to promote genes required for glial development while inhibiting those required for neuronal differentiation [36–38]. As the CNS continues to increase in size during neural development, the glial network elaborates accordingly via membrane extension, fusion and endoreplication [39–41]. Additionally, larval gliogenesis can also be generated by some neural lineages such as type II neuroblasts in the CB and a subset of neuroblasts in the OL [42, 43].

At the brain surface lies surface glia that is composed of perineural and subperineural glia [20, 33, 35]. Perineural glia lies under the CNS ECM and form a cellular meshwork that make direct contact with the circulating haemolymph and not with neural cell bodies in the brain cortex [20, 35]. Nevertheless, in a recent study, Kanai et al. suggested that perineural glia actually make physical contacts with the neuroblasts, opening up novel avenues into studying the direct communication between neuroblasts and perineural glia [30]. Perineural glia express several transporters such as the carbohydrate transporters Pippin, Major Facilitator Superfamily Transporter 3 (MSF3) and Tret1-1, allowing them to take up sugars from the haemolymph to support brain growth and functions [27, 44–46]. Beneath perineural glia are subperineural glia, which have superficial contact with neural cell bodies [20, 35]. Importantly, subperineural glia form a tight cellular network through septate junctions with each other to prevent paracellular diffusion of molecules between the haemolymph and the CNS [47–50]. Together, perineural and subperineural glia assemble the fly blood–brain barrier (BBB), which insulates the CNS. Indeed, dysregulations of septate junction proteins like Neurexin-IV (Nrx-IV) or Moody can disrupt the formation of the BBB and its integrity [48, 51]. Concomitantly, glial cells of the BBB express selective transporters such as the ABC cassette Mdr65 important for the xenobiotic responses in the fly, as well as the ECM component Col IV, although the role of Col IV in this context is so far not clear [27, 49]. Besides the structural and protective roles of the BBB, multiple studies suggested that surface glia can also non-autonomously control neuroblast behaviour during neural development. Indeed, surface glia-associated factors can coordinate development of the glial niche with neuroblast reactivation during early larval development, as well as their latter proliferation [30, 52–54].

Underneath the surface glia, cortex glia are in direct contact with neural cell bodies [20, 35]. This physical interaction between cortex glia and neural cells is enhanced during larval development when cortex glia undergo an extensive remodelling process, including membrane extension, cell division, endomitosis, endoreplication and cell fusion [40, 54, 55]. As such, in the larval CNS, cortex glia enwraps neuroblasts and their progeny, separating each neuroblast lineage into individual chambers. In line with its close contact with neural cells, cortex glia have been shown to be essential for regulating neuroblast behaviour, neuronal survival and neuronal functions [56–60]. Concomitantly, cortex glia, along with superineural glia, were previously demonstrated to protect neuroblast proliferation against oxidative stress [61].

Neuropil glia consist of ensheathing glia and astrocyte-like glia [35]. Ensheathing glia surround the outer surface of the neuropil, whereas astrocyte-like glia can infiltrate the neuropil and surround neuronal axons, dendrites and synapses [35]. Thus, neuropil glia are involved in the removal and neuronal debris during axonal pruning and homeostasis of neurotransmitters [35]. However, because neuropil glia reside at a distance from neuroblasts, there have been few studies investigating their roles in regulating neuroblast behaviour. Nonetheless, a study showed that the ablation of astrocyte-like glia can cause cortex glia infiltrating into the neuropil, suggesting that neuropil glia can inhibit morphogenesis of cortex glia to delineate the cortex-neuropil boundary [62]. Therefore, neuropil glia may indirectly regulate the proliferative properties of neuroblasts through influencing the growth and the development of other glial populations [62]. A recent study reported that quiescent neuroblasts form membrane contacts with the neuropil [63], posing an interesting possibility that the neuropil and/or neuropil glia provide signals to orchestrate the decision of the neuroblasts to remain or to exit quiescence. In summary, the *Drosophila* CNS is composed of several types of glial cells, which can play instructive roles during neurogenesis.

### The CNS tracheal system

The tracheal system is an elaborated network of air-filled tubules that performs the respiratory function in insects. Development of the tracheal system in *Drosophila* has been extensively reviewed elsewhere [64, 65]. In brief, following the specification of tracheal cells from the embryonic ectoderm, primary branches of the trachea are generated via guided cell migration that is directed towards the target tissues [64]. From the growing tips of the primary branches, secondary branches arise via cytoplasmic extension of the tip cells. In turn, at the tip of the secondary branches, tracheoles sprout and are actively involved in gas exchange of target tissues [64].

In the fly CNS, the tracheal system is composed of two primary branches: the Ganglion Branch that invades the VNC and the Cerebral Trachea that invades the brain lobes (including the CB and the OL) [19] (Fig. 1). Primary branches grow along the neuropil surface and make a U-turn, to meet and then fuse with its proximal part, establishing the anastomosing perineuropilar plexus of the CNS [19, 66]. Additionally, the trachea is embedded between the neuropil and ensheathing glia [66]. Upon genetic ablations of glial cells, the Cerebral Trachea terminal branches become larger and more elaborate compared to wildtype [66]. On that account, glial cells were suggested to play an inhibitory role on tracheal growth, although the mechanistic nature of such inhibition remains unclear. Interestingly, within three larval stages (L1–3), tracheal growth has been recently shown to promote neuroblast reactivation at the early larval stages [60]. To sum up, the CNS tracheal network not only supports gas exchange in the tissue but also interacts with both glia as well as neuroblasts in the CNS and likely plays a role in regulating neuroblast proliferative properties.

## EXTRINSIC FACTORS REGULATING NEUROBLAST QUIESCENCE AND REACTIVATION

**Figure 2:**
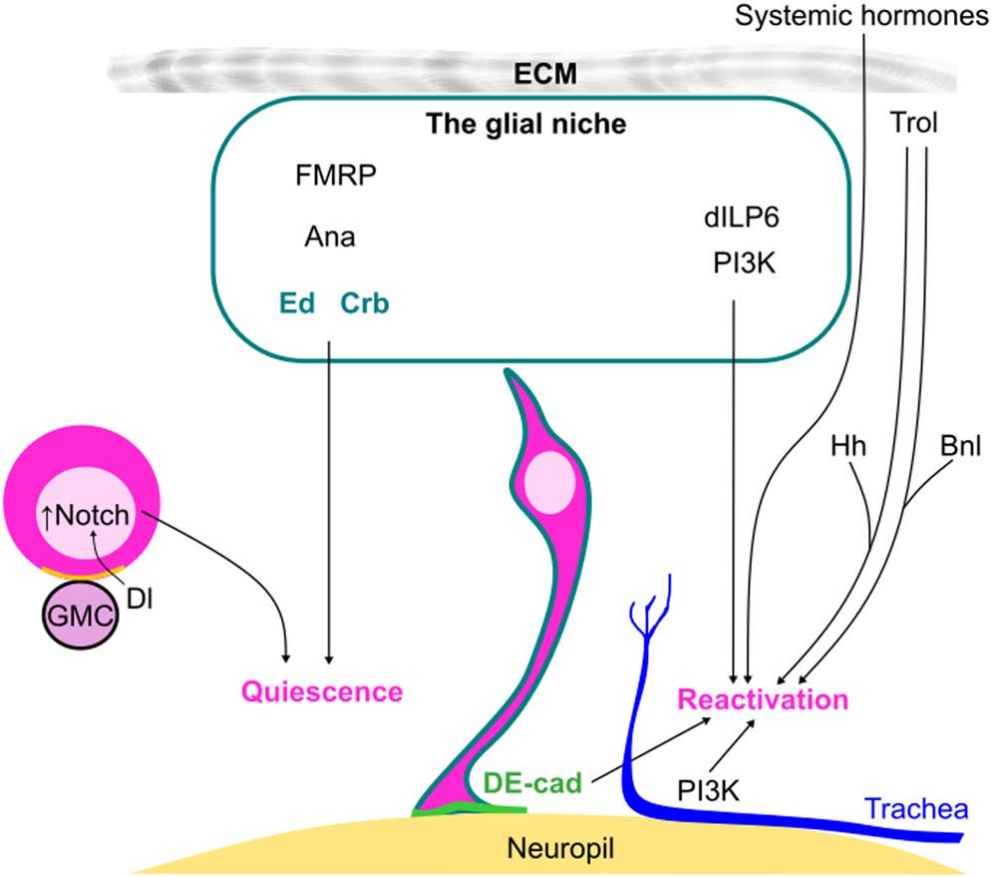
Extrinsic signals regulate the balance between neuroblast quiescence and reactivation during early larval development. The entry of neuroblasts (pink) into quiescence during late embryogenesis is partially induced by Dl/Notch signalling mediated by newborn GMCs in the proximity. The quiescent state of neuroblasts was suggested to be maintained by multiple factors derived from the glial niche such as FMRP, Ana, as well as the Ed/Crb-mediated Hippo signalling. Each quiescent neuroblast extends a primary basal protrusion that is in contact with the neuropil and is enriched with DE-cad. DE-cad in turn promotes neuroblast reactivation during early larval development. Neuroblast reactivation is induced by the release of dILP6 from glial cells, especially cortex glia, and non-autonomous upregulation of PI3K signalling in cortex glia and the trachea (blue). The extracellular matrix component Trol can promote neuroblast reactivation by upregulating the Hh and Bnl signalling cascades. Systemic hormones, such as dILP2 and Ecdysone, were also suggested to play roles in facilitating neuroblast reactivation at the early larval stages.

Neurogenesis in *Drosophila* occurs in two waves: embryonic and postembryonic neurogenesis. These two waves of neurogenesis are separated by a quiescent phase at the end of embryonic development in many neuroblast lineages of the CB and the VNC. In particular, most type I and type II neuroblasts enter quiescence characterized by cell cycle arrest, reduced cell growth and distinct morphologies whereby they extend an apical and a primary basal protrusion [10, 67–70]. The exceptions include a subset of type I neuroblasts in the VNC that switch to type 0 and terminally differentiate into postmitotic neural cells [71, 72]. In contrast, mushroom body neuroblasts and a pair of lateral neuroblasts in the CB do not become quiescent but keep proliferating throughout the embryonic-larval transition [68, 73]. During early larval development, quiescent neuroblasts are reactivated in which they re-enter the cell cycle, increase in size and lose the primary basal protrusion [67, 68, 70, 74]. Several studies have unravelled how extrinsic factors, such as dietary nutrients, humoral signalling cues and niche cells contribute to the regulation of neuroblast quiescence/reactivation [59, 60, 67, 75, 76] (Fig. 2).

### Neuroblast entry into quiescence

The entry into quiescence of neuroblasts at the end of embryogenesis has been shown to be spatiotemporally regulated by cell-intrinsic mechanisms. These include the expression of the Hox gene *antennapedia*, the dorsal patterning TF Muscle segment homeobox (Msh) the sequential expression of temporal TF (tTFs), also known as temporal patterning (Box 1) and a transient low pulse of the TF Prospero (Pros) [74, 77, 78]. However, whether and how extrinsic cues such as niche cells or systemic hormones contribute to the induction of quiescence in neuroblasts have been poorly investigated. Intriguingly, a recent study showed that during embryonic neurogenesis, proximal GMCs were proposed to facilitate the entry of neuroblasts into quiescence in a Notch-dependent manner [79]. Embryonic neuroblasts express the Notch receptor and the Notch ligand Delta (Dl) [79]. During asymmetric division of neuroblasts, Dl is unequally segregated to the GMC, which in turn activates the Notch receptor on the parental neuroblast [79]. Subsequently, as newborn GMCs continue to accumulate around the neuroblasts before terminal differentiation, Notch signalling is upregulated in the neuroblast, resulting in cell cycle exit [79]. Following terminal differentiation of the GMCs into neurons or glial cells, Dl expression is downregulated in postmitotic cells, leading to the inactivation of Notch signalling in the neuroblast that is required for their reactivation during early larval stages [79].

Box 1: CNS temporal patterning in *Drosophila.*

During development, tTFs are sequentially expressed in the aging neuroblasts [17]. The ordered expression of tTFs is required for timely neuroblast cell cycle entry, exit from quiescence, termination and the types of progenies that neuroblasts produce during neurogenesis. As such, CNS temporal patterning is instrumental to neural cell number and diversity. Temporal patterning is present in all neuroblast lineages across the CNS, with a distinct cascade of tTFs involved in each lineage. For example, embryonic type I neuroblasts express the sequence of tTFs including Hunchback (Hb) → Krüppel (Kr) → Pdm → Castor (Cas) [78, 80]. Later, postembryonic neuroblasts of type I and type II lineages express the early tTFs IGF-II mRNA binding protein (Imp)/Chronologically inappropriate morphogenesis (Chinmo)/Lin-28 and then shift to the late tTFs Syncrip (Syp)/Broad (Br)/Ecdysone-induced protein 93F (E93) [42, 81]. In type II lineages, besides neuroblasts, INPs also express a temporal cascade composing of Dicheate (D) → Grh → Eyeless (Ey) [82]. In the OL, OPC neuroblasts express the temporal cascade of Homothorax (Hth) + SoxNeuro (SoxN) + doublesex-Mab related 99B (dmrt99B) → Odd paired (Opa) → Ey + Earmuff (Erm) → Ey + Opa → Sloppy paired (Slp) + Scarecrow (Scro) → Dicheate (D) → BarH1&2 → Tailless (Tll), Gcm [43, 83]. Whereas IPC neuroblasts sequentially express Asene (Ase) → D → Atonal (Ato) → Tll → Dachshund (Dac) [13]. Temporal patterning is under the control of intrinsic mechanisms like cross-regulatory interactions, cell division or feedforward signalling, which contribute to the mutually exclusive states and unidirectional progression of the temporal series [17, 84–86]. Moreover, extrinsic cues such as the steroid hormone Ecdysone, can integrate into the intrinsic process of temporal patterning to modify the process of neurogenesis in accordance with the organismal developmental timing [42, 87–89]. In addition to the developmental context, neuroblast tumours were recently shown to redeploy temporal patterning, which specifies intra-tumour heterogeneity, tumour trajectory as well as their metabolic profiles [90]. Moreover, the temporal identities of neuroblast tumour cell-of-origin can determine the competence of tumours to acquire malignancy [81, 91–93]. On the other hand, oncogenes can inhibit temporal progression in neuroblast tumours, to prevent their timely termination, promoting tumour malignancy [94].]

### The maintenance of neuroblasts in quiescence

Unlike the entry of neuroblasts into quiescence, the decision of neuroblasts to remain quiescent or to re-enter the cell cycle during early larval development has been extensively shown to be influenced by multiple extrinsic factors, including dietary nutrients, hormonal cues and niche cells [59, 60, 67, 75]. Ebens et al. showed that a population of glial cells in the larval CNS express *anachronism* (*ana*), which encodes for a secreted glycoprotein [95]. Using mutants of *ana*, the authors showed that Ana is required to sustain the cell cycle dormancy of neuroblasts during early larval development [95]. However, the glial cell types expressing Ana remains to be deciphered. In another study, Callan et al. reported that the expression of the RNA binding protein Fragile X Mental Retardation Protein (FMRP) in glia promotes neuroblast cell cycle re-entry at the early larval stages [96]. Because FMRP mutants exhibited an elevation in Insulin signalling activity in neuroblasts that is required for their reactivation, glial FMRP was postulated to modulate the expression of Insulin-like peptide (dILP) in glial cells [97].

Glial cells and neuroblasts both express the cell–cell contact proteins Crumb (Crb) and Echinoid (Ed) during early larval development [98]. This Crb-Ed-mediated interaction between the glial niche and neuroblasts sustains neuroblast quiescence through the regulation of Hippo signalling to restrict cell growth and cell cycle progression [98–101]. Importantly, both Crb and Ed expressions are downregulated upon nutrient starvation, suggesting that Hippo signalling-mediated neuroblast reactivation lies downstream of nutrient availability [98]. Together, these data show that the glial niche can provide signalling cues to prevent precocious neuroblast reactivation and impede premature postembryonic neurogenesis in a nutrient-dependent manner.

### The reactivation of neuroblasts

During early larval development, reactivation of neuroblasts occurs in an anterior to posterior fashion, first in the CB, then VNC, followed by the abdominal region [68, 70]. Such patterning of neuroblast reactivation suggests that spatial factors such as *hox* genes play roles in regulating the timely cell cycle re-entry of neuroblasts. While how neuroblast-intrinsic machinery controls their reactivation remains poorly understood, the regulation by extrinsic signals has been extensively studied.

Early literature using mutants of the ECM component Terribly reduced OL (Trol) (the fly ortholog of mammalian Perlecan) showed that this heparan sulphate proteoglycan can promote neuroblast reactivation by controlling the expression of the cell cycle regulator Cyclin-E [29, 31]. Interestingly, Trol was also shown to form complexes with the Fibroblast Growth Factor (FGF-2) Branchless (Bnl) and Hedgehog (Hh) [31]. As Bnl and Hh signalling are required in the larvae for timely neuroblast reactivation in the early larval stages, it was proposed that Trol interacts with Bnl as well as Hh to enhance their binding to corresponding receptors on the neuroblast [31]. However, whether Hh and Bnl signalling autonomously promote neuroblast reactivation requires further assessment.

The homeobox-containing transcriptional repressor Even-skipped (Eve) can non-autonomously upregulate neuroblast reactivation during early larval development [76]. Park et al. suggested that Eve acts in the Trol pathway to control neuroblast reactivation via cell cycle regulation [76]. Concomitantly, because neuroblast reactivation can be rescued by the addition of the steroid hormone Ecdysone (Box 2) in the *eve* knockdown background, it was postulated that Eve is necessary for the synthesis of Ecdysone, which in turn promotes neuroblast cell cycle entry [76]. Alternatively, Ecdysone could also regulate the production of an Eve-dependent signal important for neuroblast exit from quiescence [76]. On that account, the relationship between Eve and Ecdysone awaits to be investigated.

One of the most well-understood environmental signals that modulates neuroblast reactivation from quiescence is that of dietary nutrients. Studies carried out more than 20 years ago showed that only in the absence of dietary amino acids but not nucleotide precursors, lipids or vitamins, which neuroblasts fail to reactivate [75]. Nonetheless, amino acids are not sufficient to induce neuroblast reactivation *ex vivo* [75]. Dietary amino acids are first sensed by the fat body of the animal [59]. This in turn leads to the production of a yet unidentified mitogen from the fat body that induces Phosphoinositol-3-Kinase (PI3K)/Target of rapamycin (TOR) signalling in the glial cells [59, 67, 75]. Subsequently, the glia secrete dILP6, which non-autonomously activates the insulin receptor (InR)/PI3K/Akt signalling cascade required for neuroblast reactivation [59, 67] (Fig. 2). Mechanistically, InR/PI3K/Akt signalling in the neuroblasts induce cell cycle progression through upregulating the cell cycle regulators Myc and Tribbles [69, 102–104]. Moreover, it can upregulate the cell growth regulators Ribosomal Protein Kinase (S6K) and Initiation Factor 4E Binding Protein (4E-BP) to facilitate protein synthesis in neuroblasts, resulting in increased cell growth [105–107]. Downstream of PI3K/TOR signalling, the spindle matrix protein Chromator (Chro) represses nuclear Pros expression in the neuroblast while inducing the expression of the TF Grainyhead (Grh) that maintains neuroblast proliferation [108, 109]. Notably, upregulation of InR/PI3K/Akt signalling in reactivating neuroblasts are coordinated with downregulation of Hippo signalling by the Striating Interacting Phosphatase and Kinase (STRIPAK) members MOB kinase activator 4 (Mob4) and Connector of kinase to AP-1 (Cka) [110].

Although *dilp* is expressed in both subperineural glia and cortex glia, its expression in the cortex glia, but not subperineural glia, is required for CB neuroblast exit from quiescence [59, 60]. But what are the functions of dILPs derived from the subperineural glia? dILP6 released from the subperineural glia layer is orchestrated by calcium oscillations that is regulated by the gap junction proteins Innexin (Inx)-1 and -2 [53]. Under nutrient scarcity, the Inx proteins are downregulated, followed by disrupted calcium oscillations and a reduction in the rate of neuroblast reactivation [53]. Therefore, robust dILP6 secretion from subperineural glia was suggested to coordinate neuroblast reactivation in different neurogenic regions approximately within 24 hours during early larval development, in response to dietary amino [53, 68, 70, 75]. Notably, the wave of neuroblast reactivation from anterior to posterior remains unchanged regardless of genetic manipulations in the glial niche [59]. Hence, it is likely that the synchronous dILP6 release from the BBB reflects the organismal nutritional status that imposes a permissive state that allows neuroblast reactivation to take place in their specific spatial order in the larval CNS. Moreover, although *dilp* overexpression in the BBB is not sufficient to induce neuroblast reactivation during early larval development, dILP6 promotes membrane expansion and chamber formation of the cortex glia that is necessary for neuroblast cell cycle re-entry [54]. This suggests that dILP6 derived from the subperineural glia may indirectly regulate neuroblast reactivation by upregulating PI3K signalling in the cortex glia. Alternatively, the glial subtypes required for neuroblast quiescence could be regulated differently in different brain regions, such as the VNC versus the CB.

Systemic dILP2, which is produced by Insulin-producing cells in the CNS, is capable of inducing PI3K signalling in the Cerebral Trachea, to trigger tracheal growth and morphogenesis in a nutrient-dependent fashion at the early larval stages [60]. In addition to its autonomous roles in tracheal development, PI3K signalling in the trachea non-autonomously promotes neuroblast reactivation in the CB [60]. It is possible that tracheolation may enhance gas exchange in the CB to promote timely neuroblast reactivation. Alternatively, the tracheal system may secrete factors that facilitate neuroblast reactivation in a paracrine manner. Nevertheless, it is of note that tracheal growth is neither affected by PI3K signalling in glial cells nor neuroblasts, suggesting that its reliance on nutrient availability for elaboration is not relayed via the glia niche but rather directly via systemic dILP levels [60].

A recent study showed that the primary basal protrusion of the quiescent neuroblasts is due to acentrosomal polymerization of microtubules in a plus-end out direction towards the neuropil [63]. This basal protrusion generates a contact site between the quiescent neuroblasts and the neuropil that is enriched with the adhesion protein DE-cadherin (DE-cad), which promotes neuroblast reactivation [63]. Because neuropil glia (including ensheathing glia and astrocyte-like glia) surround and infiltrate the fly neuropil (Fig. 1B′), it is therefore plausible that the contact between quiescent neuroblasts and the neuropil is made via these glial cell types. Moreover, it is plausible that the neuropil provides a signalling cue to promote neuroblast exit from quiescence. As such, the glial subtype involved, and the signals derived from the neuropil that regulate neuroblast reactivation remain to be identified. A recent work of Bostock et al. employing long-term live imaging showed that upon cell cycle re-entry, reactivated neuroblasts asymmetrically divide to generate the first GMC which then inherits the primary basal protrusion [74]. Nonetheless, the mechanism behind this phenomenon and its implications for neurogenesis is not yet understood.

Taken together, these studies demonstrated that extrinsic signals, including systemic cues, niche cells and their associated factors can coordinate the decision of neuroblasts to maintain or to exit quiescence and resume neurogenesis during early larval development, with the organismal nutritional status.

Box 2: The systemic hormones Ecdysone and dILPs coordinatedly modulate body and tissue growth

In insects such as *Drosophila*, tissue growth and maturation are delineated into distinct developmental stages, such that tissue growth predominantly occurs during larval development, whereas maturation takes place in the pupal stages. Accordingly, the fly final body size is determined by the timing at which the animals cease feeding and commence metamorphosis. This depends on two major factors: the growth rate of the animal, mediated by the activation of the Insulin signalling pathway via dILPs; and the growth duration, governed by the hormone Ecdysone [105]. Ecdysone is a steroid hormone produced by the prothoracic gland, which gates developmental transitions in insects. Between the three instars of larval development, pulses of Ecdysone induce larval moulting [111]. During early L3, larvae reach the critical weight (CW), a developmental checkpoint at which 50% of individuals can pupariate without further feeding [112]. The attainment of the CW is associated with a small pulse of Ecdysone that prepares tissues for feeding cessation and the initiation of metamorphosis [111, 113]. At the end of L3, a high pulse of Ecdysone triggers metamorphosis, terminating the growth phase. Notably, biosynthesis of Ecdysone is dependent on the InR/PI3K/TOR signalling activity in the prothoracic gland that is linked to the organismal nutritional status [113, 114]. Conversely, Ecdysone can inhibit TOR signalling in the fat body, to trigger the release of a yet to-be-identified factor that mediates dILP biogenesis in the insulin-producing cells [115]. Thus, dILPs integrate environmental stimuli with ecdysteroids to regulate tissue and body growth in conjunction with developmental timing of the animal.]

## EXTRINSIC FACTORS REGULATING NEUROBLAST PROLIFERATION

### Glia-associated factors

**Figure 3:**
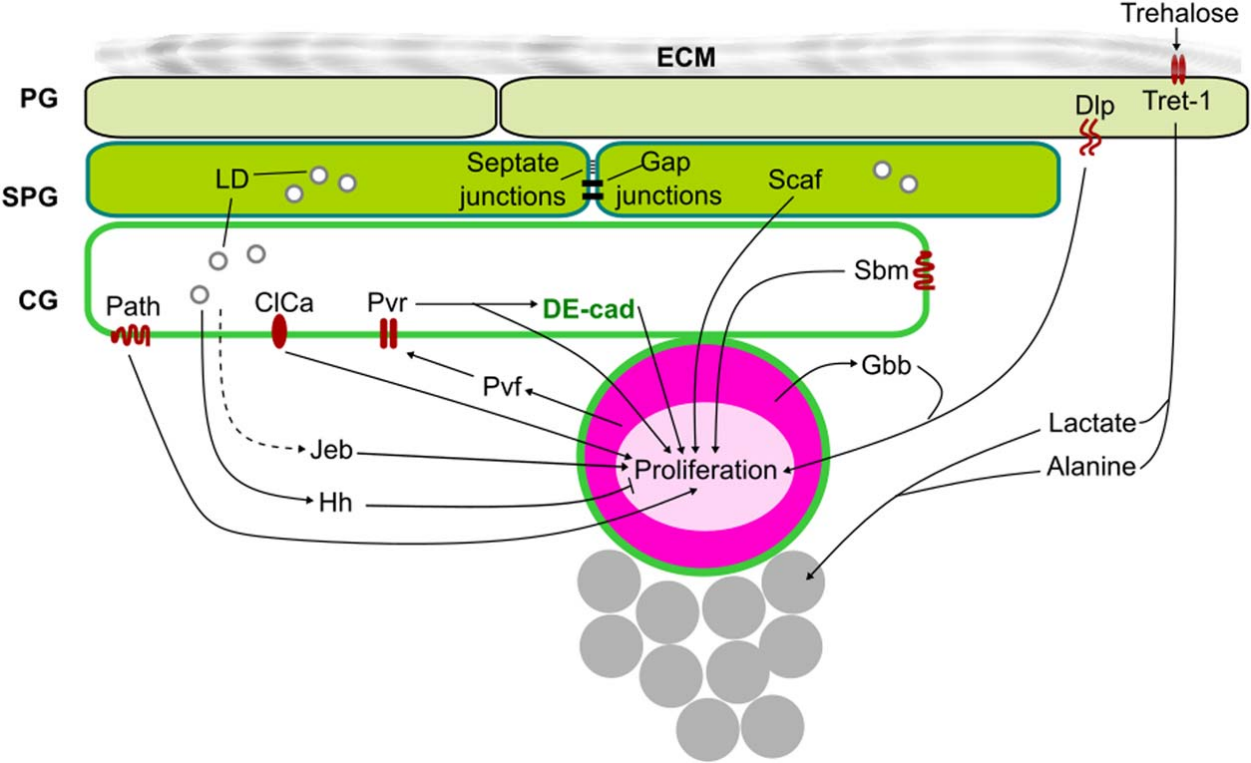
Extrinsic signals regulate neuroblast proliferation during larval development. The glial niche (green) provides multiple factors to regulate neuroblast (pink) proliferation. Perineural glia (PG) produce Dlp, which facilitates the activation of autocrine Gbb-induced TGFβ signalling in the neuroblast to promote proliferation. PG also express Tret-1 that uptakes Trehalose from the haemolymph to process it into lactate and alanine that are supplied to neurons (grey) for energy production. Subperineural glia (SPG) express Scaf while cortex glia (CG) express the amino acid transporters path, Sbm and the chloride channel ClC-a, which all promote neuroblast proliferation. The CG and the neuroblast crosstalk through Pvr/Pvf signalling in which Pvr signalling in the CG can non-autonomously upregulate PI3K signalling and DE-Cad in the neuroblast. Jeb, which can be produced by glial cells, activates Alk/PI3K signalling in the neuroblast to promote proliferation regardless of the organismal nutritional status (dashed line indicate unclear cellular source). At the late larval stages, CG produces Hh that activates Hh signalling in the neuroblasts to suppress their proliferation. CG and SPG also contain lipid droplets (LD) that can non-autonomously affect neuroblast proliferation.

Following neuroblast reactivation, the glial niche continues to support neuroblast proliferation (Fig. 3). Neuroblasts produces Glass bottom boat (Gbb), which is the fly ortholog of the mammalian bone morphogenetic protein, a ligand of the transforming growth factor β (TGFβ) signalling pathway [30]. Gbb can induce TGFβ signalling in an autocrine manner to promote neuroblast proliferation during larval neurogenesis [30]. The ability of Gbb to bind to its receptor on the neuroblast can be enhanced by the heparan sulphate proteoglycan Dally-like (Dlp) derived from perineural glia [30]. Moreover, as Dlp can act as a co-receptor for Gbb, neuroblast-derived Gbb can also activate TGFβ signalling in the perineural glia to promote proliferation of the perineural glia [30]. Together, Dlp couples development of the perineural glia layer with underlying neuroblast proliferation in the cortex. Although the authors showed that perineural glia can make direct contact with neuroblasts to regulate Dlp-mediated neuroblast proliferation, it was suggested that transcytotic transport of membrane materials including Dlp might also take place [30]. Subperineural glia express the serine protease Scarface (Scaf), which plays a key role in inhibiting BBB development, by affecting superineural glia endoreplication and perineural glia proliferation [52]. At the same time, glial Scaf promotes neuroblast proliferation in a nutrient-dependent manner [52]. Collectively, Dlp and Scaf exemplify surface glia-derived factors that autonomously regulate BBB development, and non-autonomously influence neuroblast proliferation to couple BBB development with neurogenesis.

During larval CNS, DE-Cad is expressed in glial cells, neuroblasts and a population of newborn neurons [116]. Interestingly, knock down of DE-Cad in glial cells can non-autonomously reduce neuroblast proliferation [116]. However, which glial cell types are involved in this DE-Cad-mediated regulation of neuroblast cycling remains to be addressed. Additionally, by which mechanism that glial DE-Cad influences neuroblast proliferation is also poorly understood. It is possible that factors downstream of DE-Cad signalling can regulate neuroblast proliferation. Concomitantly, DE-Cad-mediated adhesion between the glial cells and the neuroblasts may be important for neuroblast proliferation. During pupal development, DE-Cad expression in the neuroblasts was proposed to anchor the neuroblasts to their native microenvironment, likely via generating an adhesion site between the neuroblasts and cortex glia as well as daughter GMCs [117]. Intriguingly, DE-Cad expression in mushroom body neuroblasts is under the regulation of PI3K signalling [117]. When PI3K signalling is downregulated and apoptosis fails at the late pupal stages, mushroom body neuroblasts tend to migrate towards a neurogenic niche that is often populated by neurosecretory cells [117]. These results suggest that neuroblasts are trophic for growth factors, and that cues derived from the niche can instruct neuroblast behaviour, including proliferation. Notwithstanding, whether cortex glial DE-Cad involves in the positioning of mushroom body neuroblasts has not been demonstrated.

In another study, Read showed that neuroblasts express the PDGF- and VEGF-related factor (Pvf) that can bind to its receptor PDGF- and VEGF-receptor related (Pvr) in the cortex glia [57]. Pvr signalling in the cortex glia in turn autonomously upregulates DE-cad expression as well as PI3K signalling, both of which can non-autonomously promote neuroblast proliferation [57, 116]. Together, the interplay between cortex glia and neuroblasts through Pvr/Pvf signalling generates a positive feedback loop that enhances neuroblast proliferation during larval development.

Glial cells can secrete Jelly-belly (Jeb) whose expression is regulated independently of dietary nutrition [118]. Jeb binds to its receptor Anaplastic lymphoma kinase (Alk) expressed by the neuroblasts to activate PI3K/Akt signalling, which in turn promotes cell growth and proliferation, ensuring that neuroblast proliferation beyond reactivation occurs regardless of the nutritional status of the animal [118]. Furthermore, neuroblast growth is uncoupled from the amino acid-sensing pathway consisting of TOR Complex 1 (TORC1), Ras homolog enriched in brain (Rheb), and the negative regulators tuberous sclerosis complex 1 and 2 (TSC1/TSC2) [118]. Together, these mechanisms unlink neuroblast proliferation from the larval nutritional status, offering a paradigm for brain sparing—a phenomenon in which brain growth is not reduced alongside the rest of the body during human gestation upon nutrient restriction [119, 120]. However, the specific glial source of Jeb is yet to be identified. Furthermore, as Jeb is also expressed in a low level in neuroblasts as well as the surrounding neurons [118], whether it is exclusively glial Jeb that promotes neuroblast proliferation remains to be addressed.

Cortex glia express the amino acid transporter of the solute carrier (SLC) 36 family, Pathetic (Path), which is non-autonomously required for neuroblast proliferation under nutrient restriction [121]. Another amino acid transporter that is expressed in the cortex glia is the SLC7 family member Sobremesa (Sbm) [122]. Glial Sbm expression also non-autonomously promotes neural proliferation, although the underlying mechanism requires further investigation [122]. Hence, Path and Sbm present themselves to be possible candidates participating in amino acid sensing in the neurogenic niche that can potentially modulate glial Jeb production to promote neuroblast proliferation.

### Glial lipid metabolism

In the developing CNS, lipid storage organelles called lipid droplets (LDs) are specifically localized to cortex glia and subperineural glia [61, 123] (Fig. 3). LDs are composed of a neutral lipid core (e.g. triacylglyceride and sterol esters) surrounded by a phospholipid monolayer. The formation and the growth of LDs are reliant on *de novo* fatty acid synthesis and triacylglyceride generation [124, 125]. In contrast, the breakdown of LDs is catalysed by lipases and lipophagy [126, 127]. As such, the dynamics of LDs reflects the intracellular lipogenesis/lipolysis balance**.** Upon nutrient restriction, lipolysis of LDs provides cells with free fatty acid moieties that can be channelled to membrane biogenesis and oxidation for energy production [128, 129]. Thus, LDs play essential roles in the energy and lipid homeostasis of the cells.

Besides maintaining metabolic homeostasis, glial LDs play significant roles in the non-autonomous regulation of neuroblast proliferation. In the late larval stage, LD formation in cortex glia is essential for Hh-mediated crosstalk of cortex glia with neuroblasts. Indeed, inhibition of LD formation leads to a non-autonomous decrease in Hh signalling in the neuroblasts, resulting in a reduced rate of neuroblast proliferation [56]. In the cortex glia, the formation of LDs is regulated by autonomous FGF signalling [56]. FGF signalling also modulates cortex glia membrane extension as well as the expression of lipid metabolic enzymes required for Hh palmitoylation, affecting the signalling ability of the Hh ligand, and subsequently, its potential to activate Hh signalling in the neuroblasts [66, 130, 131]. Taken together, FGF signalling-mediated lipid metabolism in the cortex glia links the inhibition of neuroblast proliferation with the morphogenesis of the surrounding cortex glial niche.

LDs that have accumulated in the subperineural and cortex glia also contribute to CNS sparing under hypoxia [61]. In a poorly oxygenated environment, cellular reactive oxygen species (ROS) generation induces the peroxidation of polyunsaturated fatty acids (PUFAs) at the cell membrane, giving rise to 4-Hydroxynonenal (4-HNE), an aldehyde that can form protein adducts [132, 133]. As such, PUFA peroxidation can damage the plasma membrane, whereas 4-HNE can damage cellular proteins. Additionally, 4-HNE upregulates intracellular ROS production, creating a positive feedback loop that suppresses neuroblast proliferation [61]. Under hypoxia, the number of glial LDs increases, and they act as a defence mechanism whereby PUFA translocates from the cell membrane to cytoplasmic LDs in the glial cells [61]. This in turn prevents PUFA peroxidation at the plasma membrane, inhibiting the generation of 4-HNE and the subsequent positive feedback loop of ROS production. Therefore, LDs were deduced as an antioxidant organelle that non-autonomously protects neuroblast cell membrane and macromolecules, buffering neuroblast proliferation under oxidative stress [61].

To sum up, the formation of LDs in subperineural and cortex glia is of paramount importance for glial communication with neuroblasts. In the developing CNS, LDs modulate the signalling potential of Hh to non-autonomously affect the rate of division of the neuroblasts [56]. Furthermore, LDs can function as a protective organelle against oxidative stress, promoting brain sparing under unfavourable conditions during development [61].

## EXTRINSIC FACTORS REGULATING NEUROBLAST TERMINATION

At the end of neurogenesis, neuroblasts of different neural lineages are eliminated through distinct mechanisms. For instance, type I thoracic neuroblasts in the VNC exit the cell cycle and terminally differentiate at around 24 hours after pupal formation, whereas type I abdominal neuroblasts in the VNC are eliminated via apoptosis [68, 134, 135]. Mushroom body neuroblasts are the last to be terminated through apoptosis or autophagy during late pupal development [136, 137]. In most cases, neuroblast termination is associated with reduces in cell growth and proliferation rate, scheduled by peaks of Ecdysone during larval and pupal development [138].

Temporal patterning in the CB neuroblasts induces the expression of Ecdysone Receptor (EcR), enabling neuroblasts to respond to ecdysteroids [89]. At the CW, a pulse of Ecdysone activates Ecdysone signalling, which in turn induces a switch in the expression of two RNA-binding proteins from Imp to Syp in the neuroblasts, contributing to the generation of neural cell diversity as well as the scheduling of neuroblast terminal differentiation [42, 89]. Before the CW, Imp inhibits the activity of the Mediator complex required for neuroblast terminal differentiation [89, 138]. In addition, Imp stabilizes *myc* mRNA, leading to increased cell growth and cell division [139]. Myc also interacts with the chromatin remodelling complex Tip60 to induce the expression of atypical Protein Kinase C (aPKC), which promotes neuroblast self-renewal and inhibits differentiation [140–143]. Moreover, the Myc-Tip60 complex suppresses the nuclear localization of Pros in the neuroblast to prevent their premature cell cycle [142]. As such, extending the window of Imp expression can prolong neuroblast proliferation and delay their termination, partly via upregulating cell growth and self-renewal [89]. Conversely, while Syp does not cause neuroblast terminal differentiation *per se*, it promotes Pros nuclear accumulation to induce timely cell cycle exit of the neuroblasts [89].

After the CW, another high Ecdysone pulse at the larval-pupal transition induces a switch in the metabolic profile of the CB neuroblasts from aerobic glycolysis to oxidative phosphorylation in synchrony with an upregulation of the Mediator complex (enabled by downregulation of Imp at this stage) [138]. While glycolysis produces energy and intermediate metabolites that support rapid cycling of the neuroblasts, oxidative phosphorylation promotes metabolite depletion, resulting in the failure of the neuroblasts to regrow with each division. As such, while larval neuroblasts regrow to the original size after each division, pupal neuroblasts fail to do so, resulting in neuroblast exhaustion and terminal differentiation [138]. Nevertheless, it is of note that oxidative phosphorylation is not dispensable prior to the larval-pupal transition such that it is selectively required for the G1/S transition in the neuroblasts that in turn promotes the Imp/Syp expression shift at the CW [84].

In the mushroom body, Ecdysone coordinates the organismal developmental timing with the neuroblast-intrinsic temporal patterning to induce autophagy in the neuroblasts and hence, sensitize them for apoptosis during late pupal development [136, 137]. Mechanistically, Ecdysone induces a Imp/Syp expression shift that enhances E93 expression [137]. E93 in turn downregulates PI3K signalling in the mushroom body neuroblasts to promote autophagy as well as to cause a reduction in both cell growth and cell cycle progression, leading to their termination [137]. Nonetheless, because neuroblasts in other neural lineages also express Imp, Syp and E93, it is likely that other lineage-intrinsic factors exist to orchestrate E93-mediated termination of the mushroom body neuroblasts at the late pupal stages [42, 137]. Alternatively, differences in the niche of the mushroom body may also contribute to the timing of mushroom body neuroblast termination.

**Figure 4:**
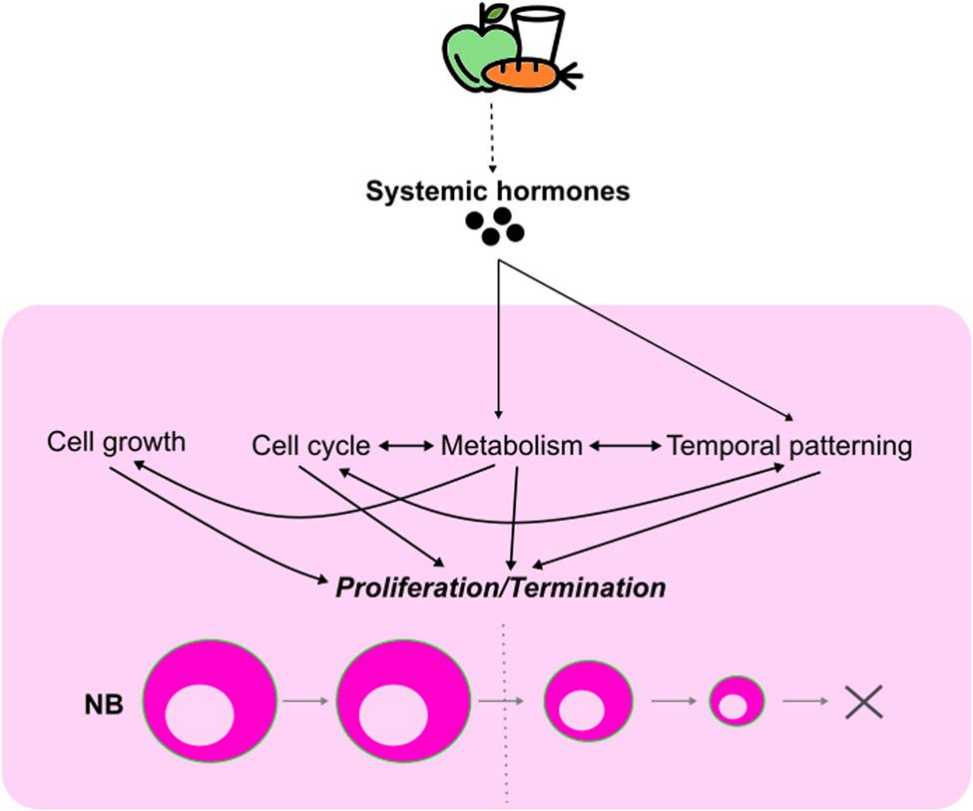
Integration of extrinsic and intrinsic cues to schedule neuroblast termination. nutrient availability induces changes in the synthesis and the secretion of systemic hormones in the animal. This influences multiple intrinsic properties of the neuroblasts, including cell growth, cell cycle controls, metabolism and temporal patterning that govern their proliferation. Additionally, intrinsic mechanisms can interact and cross-regulate each other to stimulate timely neuroblast termination.

Collectively, Ecdysone, through the induction of metabolic rewiring and temporal patterning progression, coordinates neuroblast growth, terminal division and elimination with systemic growth cessation of the animal (Fig. 4).

## EXTRINSIC FACTORS REGULATING OL DEVELOPMENT

**Figure 5:**
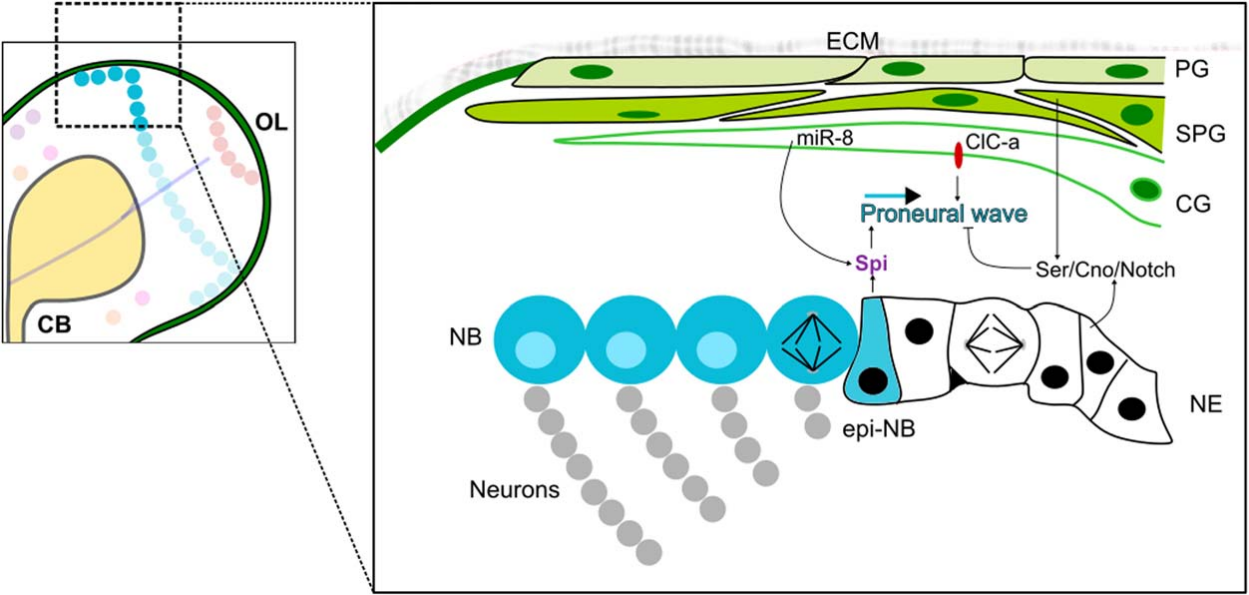
The cell types and the niche of the OL OPC in late neural development. Left: The OL during late larval development. Right: Inset showed in the left panel. In the OPC, neuroblasts (NBs, blue) are produced from the neuroepithelium (NE, black) due to a differentiating proneural wave. NE cells initially divide symmetrically to expand the progenitor pool. As the proneural wave travels across the tissue, NE cells at the wave front acquire the epi-neuroblast (epi-NB) state in which they asymmetrically divide to give rise to a NB and a ganglion mother cell (GMC). The GMC then differentiates into two neurons or glial cells of the OL. The OL glial niche (green) lies on top of the OPC, including perineural glia (PG), subperineural glia (SPG) and cortex glia (CG). The proneural wave propagation is driven by Spi secreted by epi-NBs. Spi is also produced by the CG and is directly regulated by miR-8 expression in the CG. In addition, wave propagation was suggested to be non-autonomously regulated ClC-a expression in the CG and the Ser/Cno/Notch complex formed between the SPG and the NE.

The developing OL is delineated into two main proliferative anlages: the outer and the inner proliferative centres (OPC and IPC, respectively) [144] (Fig. 1B and C). In both OPC and IPC, most neuroblasts are produced from neuroepithelial cells, starting from mid-larval development [13, 14]. However, it is of note that some neuroblasts in the OPC are also generated from neuroepithelial cells during embryogenesis [145]. In the OPC, neuroepithelial cells are differentiated into neuroblasts due to a travelling differentiation wave characterized by the expression of proneural factors such as Lethal of scute (L’sc) [146, 147] (Fig. 5). As this so-called ‘proneural wave’ propagates across the neuroepithelium, neuroepithelial cells at the wave front acquire an intermediate state between neuroepithelial cells and neuroblasts, termed epi-neuroblasts [148]. While neuroepithelial cells symmetrically divide to expand the progenitor pool, epi-neuroblasts asymmetrically divide to give rise to a neuroblast and a GMC [14, 148]. The propagation of the proneural wave is mainly driven by a travelling epidermal growth factor (EGF) Spitz (Spi) secreted by epi-neuroblasts [146, 149]. Spi then diffuses into the neuroepithelium to activate epidermal growth factor receptor (EGFR) signalling at a limited distance that upregulates the expression of proneural factors to direct the determination of neuroepithelial cells towards the neuroblast fate [146, 149]. Upon the transition, neuroblasts lose the ability to produce Spi as a feedback mechanism [146, 149]. In the neuroepithelium, activated Notch signalling maintains the neuroepithelial cell identity and thereby, in order to be differentiated into neuroblasts, Notch signalling must be turned off [150, 151]. Together, EGFR signalling coordinates with Notch signalling to modulate the speed of the proneural wave to restrict the neuroepithelial-neuroblast transition to the row of epi-neuroblasts where Notch signalling is switched off [146, 149].

The IPC can be subdivided into three regions: proximal, surface and distal IPC, in which neuroepithelial cells reside in the proximal IPC ^13^. Here, proximal IPC neuroepithelial cells generate progenitors that undergo EMT and relocate distally to another neurogenic niche—the distal IPC [13]. In this context, formation of the so-called migratory progenitors relies on the transient expression of L’sc and the spatial signalling cue Decapentaplegic (Dpp) at the margins of the IPC neuroepithelium [13]. These result in a flow of migratory progenitors from the IPC neuroepithelium towards the distal IPC, which in turn acquire the competency to differentiate into neuroblasts via the upregulation of the proneural factor Ase [13].

While the regulatory mechanisms of IPC neurogenesis remain mostly elusive, multiple studies have probed the mechanisms of neurogenesis in the OPC. Hence, in the following sections, we will focus our discussions on how extrinsic cues orchestrate different aspects of OPC neurogenesis, including neuroepithelial proliferation, their differentiation into neuroblasts and neuroblast cell cycle progression (Fig. 5).

### Regulation of OL development by Ecdysone

In the OL, the systemic Ecdysone pulse associated with the attainment of the CW marks the transition from a nutrient-sensitive to a nutrient-insensitive developmental phase of the OPC [152]. In the nutrient-sensitive phase, the expansion of OPC neuroepithelial cells is under the control of PI3K/TOR signalling [152]. However, following the Ecdysone-induced shift to the nutrient-insensitive window, neuroepithelial cells transit into neuroblasts and generate a full repertoire of neuronal subtypes regardless of the nutrient availability [152]. On that account, Ecdysone signalling was proposed to stimulate the neuroepithelial-neuroblast transition in the OPC [152].

Later studies suggested that the Ecdysone pulse associated with the CW promotes neuroepithelial-neuroblast transition by inducing the progression of temporal patterning in the neuroepithelium [87, 88]. Prior to the CW, the OPC neuroepithelium expresses Chinmo which promotes neuroepithelial self-renewal/expansion while suppressing neuroblast formation [87]. After the CW, activated Ecdysone signalling transcriptionally silences Chinmo to allow neuroepithelial-neuroblast transition to occur [87]. Concomitantly, Ecdysone signalling induces the expression of its target gene *br* that promotes neuroepithelial–neuroblast transition [88]. However, the mode of action of Chinmo and Br in the neuroepithelial-neuroblast transition is still poorly understood.

Given that the proneural wave front was observable prior to the CW [87, 146], it remains elusive how the proneural wave propagation is evoked. Interestingly, the levels of the Notch ligand Dl appear higher after than before CW, suggesting that Ecdysone may regulate Dl expression in the neuroepithelium [152]. Previous study showed that Notch hyperactivation can stall the progression of temporal patterning in type I and type II neuroblasts [94], raising an intriguing possibility that there is an integration between Ecdysone with temporal patterning through the regulation of Dl/Notch signalling in neuroepithelial cells, to permit the timely propagation of the proneural wave. Taken together, in response to the nutrient availability and developmental cues, Ecdysone stimulates the Chinmo/Br expression switch in the OPC neuroepithelium, conferring neuroepithelial cells the competence to differentiate into neuroblasts and to commence neurogenesis.

### Regulation of niche cells in OL development

Similar to the CB and the VNC, the glial niche of the OL includes perineural glia, subperineural glia and cortex glia that reside on top of the OPC (Fig. 5). Given the distinct development of the OPC, OL-associated glia have been identified with specific characteristics and functions. Subperineural glia in the OL express the Notch ligand Serrate (Ser), which forms a complex with the adherens junction protein Canoe (Cno) and the Notch receptor on the underlying neuroepithelium [153]. This Ser/Cno/Notch complex upregulates Notch signalling in neuroepithelial cells required to prevent precocious neuroepithelial-neuroblast transition [150, 151, 153]. Furthermore, as Notch signalling is a negative modulator of the proneural wave propagation, subperineural glia were suggested to non-autonomously limit the speed of the neuroepithelial-neuroblast transition [146, 149, 153]. Nonetheless, an observation that is difficult to reconcile with this model is that the OL-associated cortex glia lie between the subperineural glia and the neuroepithelium [58]. Given that the Ser activation of Notch signalling is cell contact dependent [154], it remains to be investigated how subperineural glia come into contact with the underlying neuroepithelium to suppress the neuroepithelial–neuroblast transition in the OPC by upregulating Notch signalling in the neuroepithelium.

Besides epi-neuroblasts at the wave front, the OL-associated cortex glia are another source of the Spi [58]. Before the early L3 stage, autonomous activation of EGFR signalling promotes neuroepithelial expansion, whereas afterwards it drives the propagation of the proneural wave [146]. The OL cortex glia express the microRNA miR-8, which directly inhibits Spi expression [58]. Moreover, miR-8 knockdown in the cortex glia phenocopied the misexpression of EGFR signalling in the neuroepithelium, such that the neuroepithelium appears overgrown with ectopic neuroblast generation [58, 146]. Therefore, it was postulated that there is a feedback mechanism from the neuroepithelium to the miR-8^+^ cortex glia that modulates the expression and/or the secretion of Spi, to fine tune neuroepithelial expansion and neuroepithelial–neuroblast transition [58]. Nonetheless, such a feedback mechanism is yet to be identified.

The miR-8^+^ OL-associated cortex glia also express Chloride channel-a (ClC-a) that non-autonomously promotes neuroepithelial expansion during early L3 [155]. Additionally, ClC-a expression enhances the neuroepithelial-neuroblast transition, neuroblast proliferation as well as neuronal survival during L3 [155]. However, the mechanism underlying the influence of ClC-a on OL development is still a conundrum. One hypothesis is that ClC-a can govern membrane potential of the cortex glia [155]. This is important for the regulation of intracellular calcium concentration of the cortex glia necessary for molecular secretion of pro-proliferation factors. On the other hand, it is also possible that ClC-a is involved in pH homeostasis within the niche that in turn affects neural cell proliferative properties and viability [155]. Altogether, the OL-associated cortex glia support OL development via modulating the proliferation of both the neuroepithelial and the neuroblast progenitor pools (Fig. 5).

In the larval brain lobe, the OL shows reduced trachea ramification compared to the CB [156]. In addition, the distance of the cerebral trachea to neural cells (except neuroepithelial cells in the OL) correlates with the levels of hypoxia responses in neural cells [156]. As a result, the OL was identified as the most hypoxic neurogenic region in the brain lobe [156]. Furthermore, neuroblasts also appear as the cell type expressing the highest levels of hypoxia responses [156]. In the mammalian CNS, hypoxia responses can regulate the balance between NSC self-renewal and differentiation such that hypoxia-induced glycolysis promotes NSC self-renewal and proliferation at the expense of neuronal differentiation [157]. In the fly, OL neuroblasts commence neurogenesis in the early L3 stage right after their transition from neuroepithelial cells [147, 152]. However, following differentiation from the neuroblasts, OL neurons do not mature until pupal development in which they follow an extensive migration and re-organization process into columnar units of the visual processing centre [158–160]. Therefore, the low levels of tracheolation within the OL were proposed to enable the co-existence of neuroblasts with newborn neurons during late larval development [156]. This mechanism may explain how neuronal maturation is coordinated across the OL, contributing to the appropriate assembly of the retinotopic map in the visual system.

## SUMMARY AND PERSPECTIVES

The length of time that NSCs are engaged in the cell cycle and the pace at which they divide during development defines the correct size and functions of the adult CNS. In this review, we summarized findings spanning four decades in *Drosophila*, which show that extrinsic factors derived from the microenvironment, or from distal tissues, can non-autonomously modulate NSC quiescence, reactivation, proliferation and termination, key parameters that regulate neurogenesis. Additionally, extrinsic and intrinsic cues together coordinate NSC proliferation with niche and systemic growth. Similar regulatory mechanisms involving extracellular and cellular components of the niche, as well as humoral cues and nutrients have also been showed to influence the behaviour of NSCs in the mammalian CNS [161, 162]. In spite of these insights, some knowledge gaps remain.

The ability of tissues to respond and adapt to the nutritional status of the body is utmost critical for tissue homeostasis. Unlike other tissues, the proliferative ability of neuroblasts is spared under stressful conditions, including starvation and hypoxia [61, 118]. In the adult brains, mature neurons and glial cells of the BBB are metabolically coupled such that the BBB uptakes and processes nutrients available in the haemolymph and then supplies them to neurons [45]. Furthermore, localized upregulation of carbohydrate transporters in the BBB can help to buffer neuronal survival against nutrient restriction [163]. Nevertheless, direct evidence demonstrating metabolic coupling between the BBB and neuroblasts is not yet available. During development, surface glia and cortex glia have been shown to relay the nutritional status of the animal to the neuroblasts, so that neuroblast proliferation is indirectly modulated by changes in the glial niche [59, 60, 67]. Consistent with this, single-cell sequencing showed that the transcriptomes of the glia rapidly respond to starvation [164]. Although many glia-derived factors have been reported necessary for neuroblast proliferation under nutrient restriction [52, 118, 121], their modes of actions and necessary cellular sources are elusive.

In CNS cancers namely glioblastoma and medulloblastoma, tumour progression is associated with changes in the neurogenic niche. For instance, glioma stem cells can induce astrocyte reactivation, which reciprocates to promote tumour growth and metastasis [165, 166]. Concomitantly, tumour-derived factors can perturb the permeability of the BBB, with downregulations of selective transporters and junctional proteins [167]. These facilitate cancer cells to extravasate out of the CNS [167]**.** In addition, tumours stimulate angiogenesis in the microenvironment that can provide tumours with oxygen, nutrients and a route for waste disposal [168]. Therefore, CNS tumours can disrupt the integrity of the niche to support their own growth. In *Drosophila*, many models of CNS tumours have been established such as *pros* and *brain tumour* (*brat*) loss-of-function mutations that cause dedifferentiation of GMCs and INPs, respectively, into ectopic neuroblasts that uncontrollably proliferate [9, 169–171]. Nonetheless, in these neuroblast tumour models, whether and how the neurogenic niche is altered remain to be characterized.

Although properties like temporal pattering and metabolic rewiring have revealed how intrinsic factors can control neuroblast tumour growth [17, 90, 172], less is known about the roles of extrinsic factors. During development, neuroblasts are scheduled to exit the neurogenesis program in accordance with the organismal metabolic status and developmental timing [89, 137, 138]. In the pathological state, it is still a conundrum how tumour cells can bypass such cues to proliferate beyond the normal developmental timepoints. The malignant competency of tumour neuroblasts is defined by the temporal identity of the tumour cell-of-origin such that neuroblasts in the early temporal window are more susceptible to tumour overgrowth than those in the late temporal window [81]. On the contrary, neuroblasts in the late temporal window are more likely to generate benign tumours, or in some case, are eliminated [81]. Therefore, retaining tumour neuroblasts in the early temporal identities appear necessary for tumour persistence and long-term propagation. In fact, Notch signalling activation has been shown to cause an upregulation of the tTFs Cas, Svp and Hth in the neuroblasts [94]. This then prevents the temporal progression of the tumour neuroblasts, rendering them sensitive to Notch-mediated neoplastic growth [94]. Furthermore, neuroblast tumours derived from dedifferentiation were previously shown to retain the expression of the tTFs Imp, Chinmo and Lin-28 beyond the normal expression window of the larval stages [90]. Since Imp is an upstream regulator of Myc, Mediator and Chinmo [89, 90, 139], it is plausible that Imp can suppress the Ecdysone-mediated termination of tumour neuroblasts. Altogether, the interactions between neuroblast temporal patterning and mitogenic factors highlight possible mechanisms by which CNS tumours can bypass systemic and local cues that terminate the normal neurogenesis program.

## CONCLUSION

Due to the advantages of using a range of sophisticated genetic tools, *Drosophila* neuroblasts serve as an important *in vivo* model that has provided significant mechanistic understanding of the regulation of NSC proliferative properties, which have been shown to be conserved throughout evolution. With development of additional binary expression systems such as LexA-LexOp [173], QF-QUAS [174] in conjunction with the GAL4-UAS system, as well as novel cell type specific genomic techniques such as Targeted DamID [175] and spatial transcriptomics [176], *Drosophila* is well placed to address some of the aforementioned outstanding questions. While it remains to be seen whether the extrinsic regulators of stem cell behaviour discovered in the fly can be faithfully translated to the mammalian brains, *Drosophila* has much to offer in terms of building a knowledge framework and generating new molecular insights that can be tested in the mammalian system.

## SUPPLEMENTARY MATERIAL

Supplementary material are available at *Oxford Open Neuroscience* online.

## CONFLICT OF INTEREST

None declared.

## Supplementary Material

suppl_data_kvac004
