# Supplementary material for: Non-autonomous regulation of neurogenesis by extrinsic cues: a Drosophila perspective
Source: Oxf Open Neurosci. 2022 May 4;1:kvac004. doi: 10.1093/oons/kvac004 (PMC10913833; doi:10.1093/oons/kvac004)
Supplement: suppl_data_kvac004 [file suppl_data_kvac004.zip › kvac004_REVIEWER COMMENTS oxfnsc-2022-001.docx]

REVIEWER COMMENTS

OXFNSC-2022-001 Non-autonomous regulation of neurogenesis: a Drosophila perspective

Original submission

Reviewer 1

In this review, the authors summarize recent literatures in regulation of Drosophila neurogenesis, with a focus on extrinsic cues regulating the behaviors (quiescence, reactivation, proliferation and terminal differentiation) of neural stem cells/neuroblasts. The scope of the review is appropriate and timely. The review, after proper revisions, would become potentially interesting for the field of neurogenesis.

The authors review the CNS of fruit fly and neuroblast-mediated neurogenesis in the first part of the manuscript, introduce the local niches of neuroblasts (glial niche and the CNS tracheal system) in the second part, summarize recent findings of how local niches (with nutrient-related Hippo and PI3K/TOR signaling mentioned) regulate neuroblast quiescence and reactivation during early larval development in the third part, how surface glia, cortex glia, and lipid metabolism of the glial niche regulate neuroblast proliferation in the 4th-6th parts, and how the local niche regulates development of OL medulla in the 7th part. The 8th part includes how nutrient-dependent Hippo and PI3K/TOR signaling regulate neuroblast quiescence/reactivation, and how hormonal signaling and metabolism coordinate with intrinsic temporal patterning to regulate neuroblast differentiation. The 9th part talks about how Ecdysteroids and temporal patterning regulate neuroblast generation in OL. 

This partitioning leads to a lengthy and redundant manuscript, as neuroblast-mediated neurogenesis in fly and local niches have been well reviewed before, and the logic of the manuscript is somewhat confusing. Therefore, I would recommend that the authors take the time to restructure and refine the manuscript, and focus on discussing new findings about extrinsic cue-regulated neuroblast quiescence, reactivation, proliferation and terminal differentiation. 

There are a few suggestions for the authors to consider:

1) Title: “Non-autonomous regulation of neurogenesis: a Drosophila perspective” is too broad and unclear. Based on the main text of this manuscript, maybe “Extrinsic factors-mediated non-autonomous regulation of neurogenesis: a Drosophila perspective” or something like that would be better.
2) Combine and condense the “introduction” and previous part 1.
3) Reorganize and refine previous parts 3-9, and roughly divide it into 4 parts (the subheadings should be redesigned): extrinsic signals regulate neuroblast quiescence/reactivation, extrinsic signals regulate neuroblast proliferation, extrinsic signals and temporal patterning regulate neuroblast terminal differentiation, and extrinsic signals and temporal patterning regulate OL development.
4) Combine the “Conclusion and perspectives” and “Conclusion” parts.
5) P11 line 30, “the glial niche of is also involved” should be “the glial niche is also involved”.

Reviewer 2

I commend the authors on their choice of topic, with a timely focus on non-autonomous regulation of NSC proliferative behaviours, rather than the more commonly reviewed autonomous mechanisms. The work covers well a wide range of studies and themes that had not been reviewed in such an encompassing manner. It also ends with helpful summaries of integration points between extrinsic and intrinsic mechanisms of NSC proliferation control. This valuable article will be of interest to a wide range of stem cell researchers in addition to *Drosophila* neuroscientists.

I next make suggestions towards enhancing content and form. Particularly, I highlight some points that remain open despite publications asserting one thing or another, in the spirit of critical evaluation of the data and of “spaces to be watched”.

**CONTENT Abstract**

I suggest replacing the word “hormones” by “cues” or “signals” to make it broader.

**1. NSCs in *Drosophila***

This section provides a good concise overview of types of NSCs in *Drosophila* and, in some cases, their origins. This is not the focus of the review but needs to be included as background, so it is appropriately succinct. Nonetheless, I found some discrepancies of detail, some of which could be expanded for a more balanced overview. For example, some cellular and molecular particulars are provided regarding OPC development but none regarding the IPC and none either on the origins of the majority of neuroblasts (in CB and VNC). Furthermore, a couple of molecular or mechanistic points are unclear and could be enhanced whilst still brief. One was regarding how “Notch signalling orchestrates with EGFR signalling to regulate [...]” (odd grammar as something orchestrates “something”) – but this might altogether work better if only mentioned in a later section dedicated to the OPC. Another was that the “combination of apoptosis and autophagy” that terminates MBNB divisions refers to not one or the other in different cells but a combined mechanism in all these cells and what that “combination” means.

**2. The neuroblast local niche**

This section introduces the CNS ECM and major cell-types that have been implicated in regulating neuroblast proliferation. There are appropriately succinct anatomical descriptions and light-touch mention of functional roles on neuroblasts (to be further discussed in subsequent sections).

Regarding ECM components, it would be worth mentioning that at least in the embryo, they are deposited on the surface of the CNS by macrophages. Maybe a new first subheading could be created called Extracellular matrix, referring to macrophage production (presumably chronologically first) then perineural glia production. More information on molecular components and structure would also be recommended.

1

The authors state that perineural glia do not make direct contact “with neural cell bodies”, which has been the generally accepted view. Nonetheless, using complementation of split GFP, Kanai et al*.* 2018 show that perineural glia and neuroblast membranes are actually in very close proximity. This should be referred to, either here or in a later section when referring to the role of perineural glia Dlp.

I would suggest rewriting the sentence “Moreover, many studies have shown that surface glia-derived hormones and ECM components [...]” towards more open and rigorous phrasing for a couple of reasons. First, several of the studies implicating ECM components as having a role on neuroblast behaviours (e.g., Park et al*.* 2003) have no cell-type resolution so it remains unclear if the relevant source of these components is indeed surface glia or macrophages (or some other cell type). Then, despite a few very interesting studies implicating surface-glia in neuroblast behaviours, there remain some intriguing open questions about what molecules / hormones each glial subtype(s) express that matter for those behaviours (e.g., data from Spéder and Brand, 2014 & Spéder and Brand, 2018 suggests that Ilp6 expression in subperineural glia is key for neuroblast reactivation yet Sousa-Nunes et al*.*, 2011 found that induction of dILP-6 expression from a subperineural glial driver was unable to elicit reactivation whereas it was able to do so from a pan-glial driver; on the other hand, Yuan et al*.*, 2020 propose that dILP-6 is dispensable for neuroblast reactivation and that dILP-2 is the major player towards this end). Detailed discussions would fit best in the following section but the writing in this one should leave scope for such considerations.

If quiescent neuroblasts contact neuropil glia, this could serve not only to “provide inhibitory signals against neuroblast reactivation” but the opposite. It is possible that reactivation signals could be provided by one or both of the neuropil glial cell types.

**3. The local niche regulates neuroblast quiescence and reactivation alongside neuroblast cell-intrinsic mechanisms**

In the heading for this section, I would remove the underlined as intrinsic mechanisms are not the focus of the review and it creates expectation that they might be discussed in more detail. Notwithstanding, it is absolutely fitting to mention the known intrinsic mechanisms in the first paragraph, although I would suggest leaving open the possibility that entry of neuroblasts into quiescence also has contributions of cell-non autonomous cues as at this stage we do not know if that is or not the case. “Oxygen” should be omitted here as its involvement in quiescence control has not been demonstrated.

In the third paragraph, when describing the morphology of quiescent neuroblasts, “basal and apical protrusion” sounds like they are equivalent, whereas they are very different so it would be best to make that clear providing some further description. Then, “retractions” should be replaced by “absence” as it has never been shown, albeit assumed, that they retract. There should be mention of recent imaging showing basal protrusion inheritance by GMCs (Bostock et al., 2020).

In the fourth paragraph, again, the source of the ECM component Perlecan as perineural glia should be omitted as the papers cited do not demonstrate that to be the case (so this might be best altogether left out of a paragraph on glia or else made clear that the cellular source remains undemonstrated). Furthermore, Park et al., 2003 do not show that Hh and Bnl

2

“receptors [are] localised to the neuroblasts” and should not be cited for any cell type- specific information (absent in this work).

In the fifth paragraph, a bit more information could be added by stating that dietary amino acids were the only nutrient type shown to be essential (albeit not sufficient) for neuroblast reactivation. That induction of PI3K/TOR signalling in the niche is relevant should not be written as “subperineural glia that overlay the VNC” – only one of the papers cited addressed this and employed a pan-glial driver. Subsequently, Yuan et al*.*, 2020 found that, within glial subtypes, only in cortex glia did reducing PI3K signalling impact neuroblast reactivation; and that reducing PI3K signalling in trachea had an analogous albeit milder effect. The authors of the review themselves refer to this in the seventh paragraph of this section, whose contents would be best integrated with the fifth. The final sentence could be left more open for the reasons already explained (e.g., “Subsequently, one or more types of glia secrete [note typo] Insulin-Like Peptides (dILPs), which [...]”, citing also Yuan et al., 2020).

The sixth paragraph describes synchronous calcium oscillations in the BBB. Spéder and Brand, 2014, who discovered these, suggested they in turn synchronise neuroblast reactivation. Nonetheless, neuroblasts re-emerge from quiescence in a stereotypical anteroposterior order (and no perturbation so far attempted has disturbed this, which is likely underpinned by the anteroposterior Hox code). Therefore, synchronicity of BBB calcium oscillations, appear to reflect the systemic nutritional cues upstream, acting as an essential permissive signal so that neuroblasts can reactivate in their usual temporal order (dictated by other means).

**4. Surface glia support neuroblast proliferation beyond reactivation from quiescence**

As mentioned, when discussing the role of perineural glia Dlp on neuroblast proliferation readers should be made aware that there are extensive sites of very close apposition between perineural and neuroblast membranes, unless that will have been described by then.

**5. Cortex glia regulate neuroblast proliferation
5.1. The adhesion between cortex glia and neuroblasts**

This subsection should be abolished and data on requirement for DE-Cad-mediated glial- neuroblast adhesion for neuroblast position and proliferation referred to in section 3. Dumstrei et al*.*, 2003 do state that DE-cadherin “in cortex glia” regulates neuroblast proliferation but the gcm-GAL4 driver they used is expressed in all glia and, in fact, the authors themselves show that levels are highest in surface glia. It thus remains unclear what glial subtype(s) mediate this effect. Doyle et al., 2017 is suitably discussed alongside the above but also do not provide data demonstrating that it is cortex glia DE-Cad what is at play for MBNB position.

**5.2. Cortex glia secrete signalling molecules to regulate neuroblast proliferation**

In light of comments above, the first sentence should not start with “Besides adhesion [...] also”. Similarly to prior comments, Cheng et al., 2010 did not go into whether there is a glial

3

subtype where Jeb expression matters most to support neuroblast proliferation and, although Jeb expression is highest in glia, it is also significant in neurons as well as neuroblasts themselves. One option could be to merge sections 4 and 5 in order not to suggest that the roles and molecules described have been very well established as pertaining to certain glial subtype(s). This itself should be discussed. There could be paragraphs dedicated to glial subtypes glia where that has been demonstrated (e.g., content of what is currently section 5.3).

**5.3. Non-secretable factors of cortex glia regulate neuroblast proliferation**

The sentence “Perturbations in Smb expression produce larvae with increased weight due to a delay in larval-pupal transition” feels “out of the blue” and breaks the flow – I suggest omitting it.

**7. The local niche regulate development of the OL medulla**

**7.1. The OL-associated glial niche regulates proliferation of both the neuroepithelial and neuroblast pools**

By the time Spi and the proneural wave are reintroduced in this section, the reader may not remember the summary from section 1 so at least some of the molecular detail might work best if introduced only here.

**7.2 The trachea is underdeveloped in the OL**

The authors may consider changing the title of this heading as presumably the OL trachea is as developed as it “should be” (e.g. “Modest ramification of OL trachea supports extended neural stem cell proliferation”).

The first sentence here could be refined. Baccino-Calace et al., 2020 state that “the hypoxia response of individual cells correlates with their position in the brain in relation to the nearest airways”, also that “Neural stem cells in both compartments [CB and OL] show the strongest hypoxia response”, and intriguingly that NE were an exception in that they “appeared to exhibit the same oxygenation levels regardless of their distance to tracheoles”.

**8. Integration of extrinsic signals with the neuroblast intrinsic proliferation program**

In Figure 5, depiction of oxygen levels as regulating neuroblast reactivation should be removed as that has not been demonstrated.

For a complete account of neural progenitor behaviours known to be regulated by tTFs, Box 2 should indicate that the temporal state of neuroblasts also defines whether tumour- suppressor mutations result in malign neoplasia versus benign hyperplasia (Narbonne- Reveau et al., 2016; Shaw et al., 2018) and that, reciprocally, tumour suppressor mutations or oncogene expression arrest temporal progression (Zacharioudaki et al., 2015; Narbonne- Reveau et al., 2016).

4

**8.2. Hormonal signalling, temporal patterning, and metabolism regulate neuroblast terminal differentiation**

I believe the two paragraphs on this section, both of which mention Ecdysone signalling and OxPhos, could be written in a more integrated manner. A new paragraph comes as a surprise since OxPhos and catabolism had already been referred to in this context, whereas readers might expect a new concept.

**8.3. Ecdysteroids and temporal patterning in the generation of neuroblasts in the OL**

It could be interesting to refer here to Zacharioudaki et al., 2015, who found that hyperactivation of Notch signalling can arrest temporal progression in type I and II neuroblasts. Although the OL presents a distinct cellular context, this raises the hypothesis of mechanistic integration between Notch signalling levels, proneural wave propagation and temporal transitions in the OL.

**Conclusions and perspectives**

**2. Integrations of intrinsic and extrinsic factors in the regulations of CNS tumour growth**

Worth mentioning here also that BBB disruptions are found in the context of neural tumours.

Regarding the final sentence of this section, note that some of what has been reviewed explains some aspects of the “bypassing” since retention of early temporal factors in a fraction of tumour NB-like cells will stabilise Myc and preclude eliciting Mediator.

5

**LANGUAGE AND FORMALITIES**

**Abstract**

Line 11: would be better as “progenitors that in turn generate differentiated neurons” * Lines 21 and 27: remove “the”

**Introduction**

Line 26: refers to the case in mammals so that might be worth making explicit; Furthermore, in that case, do they all first divide symmetrically?
Line 35: “neuronal” should be “neural” to encompass glial cell types
Line 37: missing comma after “NPs” (changes the meaning slightly)

Line 52: worth adding “neurogenesis in *Drosophila*.” at the end of ongoing sentence **1. NSCs in *Drosophila***

Figure 1C: looks like there are NBs in the neuropil; furthermore, note that the acronym “NB” has not been introduced

Page 3 Line 43: best to say that the GMC divides, often asymmetrically, to generate two differentiated neurons or glial cells (similar to *)

Page 3 Line 49: “upon the induction of dietary nutrients” is unclear
Page 4 Line 4: Similar to * (generation of differentiated progeny rather than “differentiate”)

Page 4 Line 8: would be better as “a pair of lateral neuroblasts” (as “lateral neuroblasts alone could refer to any number)

Page 4 Line 15: make clear that INPs also divide asymmetrically

Page 4 Line 20: again should be “neural” rather than “neuronal” as some glia is produced by at least one of the type II lineages

**2. The neuroblast local niche**

Heading: by definition a stem cell niche is “local” so would remove that word, which feels redundant

Page 6 Line 15: I suggest “including various types of neurons and glia, as well as tracheal cells”

Page 6 Line 15: missing comma before “comprised”

Page 6 Line 44: again should be “neural” to be more encompassing (including neuroblasts, neurons and other glia)

6

**3. The local niche regulates neuroblast quiescence and reactivation alongside neuroblast cell-intrinsic mechanisms**

Page 14 Line 27 and Figure 3: “secret” should be “secrete”
Page 11 Line 35: “Because neuroblast exit” more elegant than ”neuroblasts’ exit”

**4. Surface glia support neuroblast proliferation beyond reactivation from quiescence**

Page 13 Line 10: first appearance of the acronym TGFb so it should be spelt out here rather than in Line 15

**5. Cortex glia regulate neuroblast proliferation**

Page 14 Line 31: “secret” should be “secrete”

**6. Lipid metabolism of the glial niche regulates neuroblast proliferation**

Page 16 Line 13: “On the contrary” would read better as “Conversely”
Page 16 Line 27: “have been known” would read better as “have been shown”

Page 16 Line 32: “essential for the Hh” should be “essential for Hh” as this has not yet been referred to

Page 17 Line 6: “poor” should be “poorly”
Page 17 Line 7: “Acid (PUFA)” should be “Acids (PUFAs)” as there are multiple Page 17 Line 22: again PUFAs – should be plural

**7. The local niche regulate development of the OL medulla**

Typo in heading: should be “regulates”

Page 18 Line 5: instead of “division”, “development” would be preferable

Page 18 Line 13: “neuroepithelial” should be “neuroepithelium (NE)”

Page 18 Line 18: “, which” should be deleted; and “preventing precocious neuroblast transition” would be clearer as “preventing their precocious transition into neuroblasts”

Page 18 Line 34: “investigated how” could be “investigated if and how” Page 18 Line 46: “autonomously” would be better as “directly”

Page 19 Line 11: “express the chloride channel ClC-a” should be “express Chloride Channel A (ClC-a)”

**8. Integration of extrinsic signals with the neuroblast intrinsic proliferation program**

8.1 heading: better grammar would be “Nutrient-dependent Hippo and PI3K/Akt signalling coordinate neuroblast quiescence/reactivation”

7

Page 20, Line 55: “coordinately orchestrated” is redundant as “coordination” and “orchestration” convey the same message

Page 21 Line 10: “On the contrary” would read better as “In contrast”
Page 21 Line 15: “Ribosomal Protein Kinase” should be “Ribosomal Protein S6 Kinase”

Page 22 Line 6: I would reverse the order to “During neuroblast terminal differentiation (also known as decommissioning)” since “termination” is the nomenclature used up to then in the article.

Page 22: note that some text was not visible in main section and Box 2

Box 1: comma missing after DILPs, “the growth” would read better as just “growth”, “growth transition” and “prepares tissue” should be pluralised (“transitions”, “tissues”)

Page 23 Line 13: “at the end of neurogenesis” is unnecessary (redundant) Page 23 Line 31: “of neuroblasts” is unnecessary (redundant)

Page 23 Line 55: typo in “energic” which should be “energetic”, although “metabolic” might be best

9.3 Heading: should be 8.3

Page 24 Line 32 and Page 25 Line 13: “differentiate” might be better if replaced by “transition” for consistency with prior sections and to avoid confusion with terminal differentiation

**Conclusions and perspectives**

Heading: Given that a subsequent section is entitled “Conclusion” the title of this one could be edited to “Summary and perspectives”

Subheadings numbered herein as 1 and 2 should be avoided given this numbering already used for article sections (distinct paragraphs without titles might suffice)

Page 15 Line 24: instead of “recent” it would do the authors more justice to say “we summarised data spanning four decades of findings that show”

Page 25 Line 29: I suggest deleting “the” as a key parameter that regulates neurogenesis not discussed as regulated by extrinsic cues was asymmetric division (understandably, given limited evidence to this effect but “the” sounds as if those listed constitute all the key parameters)

Page 25 Line 33: would read better as “NSC proliferation with niche and systemic growth”

**References**

Reference callouts in text: preferable in chronological order
Pages numbers are missing in a few and I notice a spurious “%” sign

Decision letter

13-Feb-2022 

Dear Dr. Cheng, 

Manuscript ID OXFNSC-2022-001 entitled "Non-autonomous regulation of neurogenesis: a Drosophila perspective" which you submitted to the Oxford Open Neuroscience, has been reviewed.  The comments of the reviewer(s) are included at the bottom of this letter. 

The reviewer(s) have recommended publication, but also suggest some major revisions to your manuscript. Therefore, I invite you to respond to the reviewer(s)' comments and revise your manuscript. Please submit your revision in one month. 

To revise your manuscript, log into https://mc.manuscriptcentral.com/oxfnsc and enter your Author Centre, where you will find your manuscript title listed under "Manuscripts with Decisions."  Under "Actions," click on "Create a Revision."  Your manuscript number has been appended to denote a revision. 

You may also click the below link to start the revision process (or continue the process if you have already started your revision) for your manuscript. If you use the below link you will not be required to login to ScholarOne Manuscripts. 

*** PLEASE NOTE: This is a two-step process. After clicking on the link, you will be directed to a webpage to confirm. *** 

https://mc.manuscriptcentral.com/oxfnsc?URL_MASK=ee70ddc8f1b24d52b63593b62e112b65 


You will be unable to make your revisions on the originally submitted version of the manuscript.  Instead, revise your manuscript using a word processing program and save it on your computer.  Please also highlight the changes to your manuscript within the document by using the track changes mode in MS Word or by using bold or colored text. 

Once the revised manuscript is prepared, you can upload it and submit it through your Author Centre. 

When submitting your revised manuscript, you will be able to respond to the comments made by the reviewer(s) in the space provided.  You can use this space to document any changes you make to the original manuscript.  In order to expedite the processing of the revised manuscript, please be as specific as possible in your response to the reviewer(s). 

IMPORTANT:  Your original files are available to you when you upload your revised manuscript.  Please delete any redundant files before completing the submission. 

Because we are trying to facilitate timely publication of manuscripts submitted to the Oxford Open Neuroscience, your revised manuscript should be uploaded as soon as possible.  If it is not possible for you to submit your revision in a reasonable amount of time, we may have to consider your paper as a new submission. 

Once again, thank you for submitting your manuscript to the Oxford Open Neuroscience and I look forward to receiving your revision. 

Sincerely, 
Dr. Hongyan Wang 
Senior Editor, Oxford Open Neuroscience 
hongyan.wang@duke-nus.edu.sg

Revised submission

Author response

We thank the reviewers for their insightful comments, we have made changes according to these comments as below: 

Reviewer 1 

This partitioning leads to a lengthy and redundant manuscript, as neuroblast-mediated neurogenesis in fly and local niches have been well reviewed before, and the logic of the manuscript is somewhat confusing. Therefore, I would recommend that the authors take the time to restructure and refine the manuscript, and focus on discussing new findings about extrinsic cue-regulated neuroblast quiescence, reactivation, proliferation and terminal differentiation. 

We have reorganised the manuscript such that it focuses more on extrinsic signals regulating different behaviour of neuroblasts as suggested. 

There are a few suggestions for the authors to consider: 

1) Title: “Non-autonomous regulation of neurogenesis: a Drosophila perspective” is too broad and unclear. Based on the main text of this manuscript, maybe “Extrinsic factors-mediated non-autonomous regulation of neurogenesis: a Drosophila perspective” or something like that would be better. 

We have changed the title into “Non-autonomous regulation of neurogenesis by extrinsic cues: a Drosophila perspective” 

2) Combine and condense the “introduction” and previous part 1. 

We have combined the Introduction with section 1 as suggested. Thus, in the introduction, we now introduce the different types of neuroblasts in Drosophila in terms of their origins and modes of division. Information about their behaviour such as quiescence, reactivation and termination has been moved to their corresponding sections 2,4 and 5, right before discussing different extrinsic factors regulating this behaviour. 

3) Reorganize and refine previous parts 3-9, and roughly divide it into 4 parts (the subheadings should be redesigned): extrinsic signals regulate neuroblast quiescence/reactivation, extrinsic signals regulate neuroblast proliferation, extrinsic signals and temporal patterning regulate neuroblast terminal differentiation, and extrinsic signals and temporal patterning regulate OL development. 

We have reconstructed section 3-9 as recommended. The new sections 2-5 now become: 
2. Extrinsic factors regulating neuroblast quiescence and reactivation 
3. Extrinsic factors regulating neuroblast proliferation 
4. Extrinsic factors regulating neuroblast termination 
5. Extrinsic factors regulating optic lobe development 

4) Combine the “Conclusion and perspectives” and “Conclusion” parts. 

We have changed the heading “Conclusion and perspectives” into “Summary and perspectives” to better describe the content of the section. Hence, the final Conclusion can be maintained. 

5) P11 line 30, “the glial niche of is also involved” should be “the glial niche is also involved”. 

We have corrected this. 
  
Reviewer 2 

I commend the authors on their choice of topic, with a timely focus on non-autonomous regulation of NSC proliferative behaviours, rather than the more commonly reviewed 
autonomous mechanisms. The work covers well a wide range of studies and themes that had not been reviewed in such an encompassing manner. It also ends with helpful summaries of integration points between extrinsic and intrinsic mechanisms of NSC proliferation control. This valuable article will be of interest to a wide range of stem cell 
researchers in addition to Drosophila neuroscientists. 
I next make suggestions towards enhancing content and form. Particularly, I highlight some points that remain open despite publications asserting one thing or another, in the spirit of critical evaluation of the data and of “spaces to be watched”. 

CONTENT 
Abstract 
I suggest replacing the word “hormones” by “cues” or “signals” to make it broader. 

We have changes ‘hormones’ into ‘signals’ as suggested. 

1. NSCs in Drosophila 
This section provides a good concise overview of types of NSCs in Drosophila and, in some cases, their origins. This is not the focus of the review but needs to be included as
background, so it is appropriately succinct. Nonetheless, I found some discrepancies of detail, some of which could be expanded for a more balanced overview. For example, some 
cellular and molecular particulars are provided regarding OPC development but none regarding the IPC and none either on the origins of the majority of neuroblasts (in CB and 
VNC). Furthermore, a couple of molecular or mechanistic points are unclear and could be enhanced whilst still brief. One was regarding how “Notch signalling orchestrates with EGFR signalling to regulate […]” (odd grammar as something orchestrates “something”) – but this might altogether work better if only mentioned in a later section dedicated to the OPC. We decided to dedicate section 5 to discuss about OL development. In the foreword, we briefly introduce about the OL development in which we have expanded the part about the IPC. We have also refined how the proneural wave propagation was described. “Notch signalling orchestrates with EGFR signalling to regulate […]” has been corrected to “EGFR signalling coordinates with Notch signalling […]”. Another was that the “combination of apoptosis and autophagy” that terminates MBNB divisions refers to not one or the other in different cells but a combined mechanism in all these cells and what that “combination” means. 

Apoptosis and autophagy have been demonstrated to occur in MBNBs during late pupal development. Moreover, autophagy was suggested to sensitize MBNBs for apoptosis, even though whether this is the case remains elusive. Thus, it is uncertain whether autophagy and apoptosis take place in the same set of MBNBs is unclear. As such, we changed the wording into “apoptosis or autophagy” to avoid confusion.  

2. The neuroblast local niche 
This section introduces the CNS ECM and major cell-types that have been implicated in regulating neuroblast proliferation. There are appropriately succinct anatomical descriptions 
and light-touch mention of functional roles on neuroblasts (to be further discussed in subsequent sections). Regarding ECM components, it would be worth mentioning that at least in the embryo, they are deposited on the surface of the CNS by macrophages. Maybe a new first subheading could be created called Extracellular matrix, referring to macrophage production (presumably chronologically first) then perineural glia production. More information on molecular components and structure would also be recommended. 

We have added a new subsection 1.1 to briefly introduce the CNS ECM in terms of its components, origin and its general function in CNS shaping and neurogenesis. 
Although transcriptomic analysis of surface glia showed that they do express col IV, this was done in the adult stage, and functional tests have not been done. By contrast, manipulation of ECM components in the fat body can lead to a reduction in ECM component expressions in the CNS. As such, we think the CNS ECM, similar to other tissues, is produced by the fat body during larval development. Regarding surface glia, while they can produce factors that remodel the ECM to accommodate CNS growth, it remains plausible that they contribute to the production of ECM components given that manipulations in the fat body could not totally deplete ECM in the CNS (mentioned in section 1.2). 

The authors state that perineural glia do not make direct contact “with neural cell bodies”, which has been the generally accepted view. Nonetheless, using complementation of split 
GFP, Kanai et al. 2018 show that perineural glia and neuroblast membranes are actually in very close proximity. This should be referred to, either here or in a later section when 
referring to the role of perineural glia Dlp. 

We have incorporated this information into section 1.2 as suggested. 

I would suggest rewriting the sentence “Moreover, many studies have shown that surface glia-derived hormones and ECM components […]” towards more open and rigorous 
phrasing for a couple of reasons. First, several of the studies implicating ECM components as having a role on neuroblast behaviours (e.g., Park et al. 2003) have no cell-type resolution so it remains unclear if the relevant source of these components is indeed surface glia or macrophages (or some other cell type). 

We have removed this information in section 1.2 (introduction about the glial niche). Instead, we discuss the function and the origin of Trol/Perlecan in section 2.3. 

Then, despite a few very interesting studies implicating surface-glia in neuroblast behaviours, there remain some intriguing open questions about what molecules / hormones each glial subtype(s) express that matter for those behaviours (e.g., data from Speder and Brand, 2014 & Speder and Brand, 2018 suggests that Ilp6 expression in subperineural glia is key for neuroblast reactivation yet Sousa-Nunes et al., 2011 found that induction of dILP-6 expression from a subperineural glial driver was unable to elicit reactivation whereas it was able to do so from a pan-glial driver; on the other hand, Yuan et al., 2020 propose that dILP6 is dispensable for neuroblast reactivation and that dILP-2 is the major player towards this end). Detailed discussions would fit best in the following section but the writing in this one should leave scope for such considerations. 

We have modified section 2.3 discussing dILP derived from different glial source and their roles in neuroblast reactivation. We agree that even though both subperineural and cortex glia express dilp, only that of the cortex glia is essential for NB reactivation. On the other hand, dILPs derived from subperineural glia still plays a role in NB reactivation such that it relays the larval nutritional status with the coordinated NB reactivation across the CNS. Furthermore, dILPs from the BBB also coordinates the cortex glia chamber formation with NB reactivation. 

If quiescent neuroblasts contact neuropil glia, this could serve not only to “provide inhibitory signals against neuroblast reactivation” but the opposite. It is possible that reactivation signals could be provided by one or both of the neuropil glial cell types. 

We have corrected this. 

3. The local niche regulates neuroblast quiescence and reactivation alongside neuroblast cell-intrinsic mechanisms 
In the heading for this section, I would remove the underlined as intrinsic mechanisms are not the focus of the review and it creates expectation that they might be discussed in more detail. Notwithstanding, it is absolutely fitting to mention the known intrinsic mechanisms in the first paragraph, although I would suggest leaving open the possibility that entry of neuroblasts into quiescence also has contributions of cell-non autonomous cues as at this stage we do not know if that is or not the case. “Oxygen” should be omitted here as its 
involvement in quiescence control has not been demonstrated. 

Since we have changed the organisation of the manuscript, we chose to dedicate section 2 specifically to discuss extrinsic signals regulating neuroblast quiescence and reactivation. Here, we subdivided the section into three parts: 
2.1. The entry of neuroblasts into quiescence: Many studies have demonstrated that NB entry into quiescence is regulated by the intrinsic machinery. However, a recent study has provided an interesting insight into how the progeny surrounding neuroblasts can promote their cell cycle exit. 
2.2. The maintenance of neuroblasts in quiescence 
2.3. The reactivation of neuroblasts 
Oxygen has been omitted in the foreword as recommended. 

In the third paragraph, when describing the morphology of quiescent neuroblasts, “basal and apical protrusion” sounds like they are equivalent, whereas they are very different so it would be best to make that clear providing some further description. Then, “retractions” should be replaced by “absence” as it has never been shown, albeit assumed, that they 
retract. There should be mention of recent imaging showing basal protrusion inheritance by GMCs (Bostock et al., 2020). 

We have specified that a quiescent neuroblast has two membrane protrusions: an apical protrusion and a primary basal protrusion. We also mentioned the study of Bostock et al., 2020 as recommended, regarding that the daughter GMCs inheriting the primary basal protrusion following neuroblast reactivation. 

In the fourth paragraph, again, the source of the ECM component Perlecan as perineural glia should be omitted as the papers cited do not demonstrate that to be the case (so this might be best altogether left out of a paragraph on glia or else made clear that the cellular source remains undemonstrated). Furthermore, Park et al., 2003 do not show that Hh and Bnl receptors [are] localised to the neuroblasts” and should not be cited for any cell type specific information (absent in this work). 

We have removed the source of Perlecan/Trol, we now only mention that it is an ECM component. We agree that there is no evidence of Hh and Bnl receptor expressions in the NBs during early larval development. Thus, we have removed the information indicating that Trol forms complex with Hh and Bnl to upregulate the corresponding signalling cascade in the neuroblast, to promote neuroblast reactivation. Additionally, we have noted that it is unclear whether autonomous Hh and Bnl signalling have roles in neuroblast reactivation. 

In the fifth paragraph, a bit more information could be added by stating that dietary amino acids were the only nutrient type shown to be essential (albeit not sufficient) for neuroblast 
reactivation. 

We have added this information into section 2.3 

That induction of PI3K/TOR signalling in the niche is relevant should not be written as “subperineural glia that overlay the VNC” – only one of the papers cited addressed this and employed a pan-glial driver. Subsequently, Yuan et al., 2020 found that, within glial subtypes, only in cortex glia did reducing PI3K signalling impact neuroblast reactivation; and that reducing PI3K signalling in trachea had an analogous albeit milder effect. The authors of the review themselves refer to this in the seventh paragraph of this section, whose contents would be best integrated with the fifth. The final sentence could be left more open for the reasons already explained (e.g., “Subsequently, one or more types of glia secrete [note typo] Insulin-Like Peptides (dILPs), which […]”, citing also Yuan et al., 2020). 

We have modified section 2.3 as suggested (paragraph 3-5). 
In paragraph 3, we discuss how PI3K signalling in glia cells that induces neuroblast reactivation. 
In paragraph 4, we discuss different functions of dILP/PI3K signalling of cortex glia and subperineural glia in neuroblast reactivation. 
In paragraph 5, we discuss how systemic dILP2 affects neuroblast reactivation through the induction of PI3K signalling in glia and the trachea. 

The sixth paragraph describes synchronous calcium oscillations in the BBB. Speder and Brand, 2014, who discovered these, suggested they in turn synchronise neuroblast 
reactivation. Nonetheless, neuroblasts re-emerge from quiescence in a stereotypical anteroposterior order (and no perturbation so far attempted has disturbed this, which is likely underpinned by the anteroposterior Hox code). Therefore, synchronicity of BBB calcium oscillations, appear to reflect the systemic nutritional cues upstream, acting as an essential permissive signal so that neuroblasts can reactivate in their usual temporal order (dictated by other means). 

We have added the information regarding the anterior-to-posterior wave of neuroblast reactivation into the first paragraph of section 2.3. In the fourth paragraph of this section, we have also added a sentence regarding that synchronous dILP6 secretion from the BBB might be permissive to neuroblast reactivation in their usual spatial order as suggested. 

4. Surface glia support neuroblast proliferation beyond reactivation from quiescence 
As mentioned, when discussing the role of perineural glia Dlp on neuroblast proliferation readers should be made aware that there are extensive sites of very close apposition 
between perineural and neuroblast membranes, unless that will have been described by then. 

The close contact between NBs and perineural glia has been addressed in section 1.2. 

5. Cortex glia regulate neuroblast proliferation 
5.1. The adhesion between cortex glia and neuroblasts 
This subsection should be abolished and data on requirement for DE-Cad-mediated glial neuroblast adhesion for neuroblast position and proliferation referred to in section 3. 
Dumstrei et al., 2003 do state that DE-cadherin “in cortex glia” regulates neuroblast proliferation but the gcm-GAL4 driver they used is expressed in all glia and, in fact, the authors themselves show that levels are highest in surface glia. It thus remains unclear what glial subtype(s) mediate this effect. Doyle et al., 2017 is suitably discussed alongside the above but also do not provide data demonstrating that it is cortex glia DE-Cad what is at play for MBNB position. 

We agree that the cellular resolution DE-Cad that can regulate neuroblast proliferation is still lacking. Furthermore, it is uncertain that DE-Cad in glial cells regulate NB proliferation in an adhesion-mediated mechanism. Thus, we modified the paragraph not to imply that it is exclusively DE-Cad-mediated adhesion between neuroblasts and glial cells that can regulate NB proliferation. Hence, regulation of neuroblast proliferation in this case can also be mediated via signalling downstream of DE-Cad in NBs. We also removed any information implying that cortex glia and MBNBs have contact sites enriched of DE-Cad due to the lack of evidence in the work of Doyle et al., 2017.  

5.2. Cortex glia secrete signalling molecules to regulate neuroblast proliferation 
In light of comments above, the first sentence should not start with “Besides adhesion […] also”. Similarly to prior comments, Cheng et al., 2010 did not go into whether there is a glial subtype where Jeb expression matters most to support neuroblast proliferation and, although Jeb expression is highest in glia, it is also significant in neurons as well as 
neuroblasts themselves. One option could be to merge sections 4 and 5 in order not to suggest that the roles and molecules described have been very well established as pertaining to certain glial subtype(s). This itself should be discussed. There could be paragraphs dedicated to glial subtypes glia where that has been demonstrated (e.g., content 
of what is currently section 5.3). 

We have merged these two sections into section 3.1. (glia-associated factors regulating neuroblast proliferation). 

5.3. Non-secretable factors of cortex glia regulate neuroblast proliferation 
The sentence “Perturbations in Smb expression produce larvae with increased weight due to a delay in larval-pupal transition” feels “out of the blue” and breaks the flow – I suggest 
omitting it. 

We have omitted this sentence. 

7. The local niche regulate development of the OL medulla 
7.1. The OL-associated glial niche regulates proliferation of both the neuroepithelial and 
neuroblast pools 
By the time Spi and the proneural wave are reintroduced in this section, the reader may not remember the summary from section 1 so at least some of the molecular detail might work best if introduced only here. 

We have put this information in subsection 5.1 that gives a fundamental background on the development of the OL, prior to discussing the different extrinsic factors regulating developmental processes in this tissue. 

7.2 The trachea is underdeveloped in the OL 
The authors may consider changing the title of this heading as presumably the OL trachea is as developed as it “should be” (e.g. “Modest ramification of OL trachea supports extended neural stem cell proliferation”). The first sentence here could be refined. Baccino-Calace et al., 2020 state that “the hypoxia response of individual cells correlates with their position in the brain in relation to the nearest airways”, also that “Neural stem cells in both compartments [CB and OL] show the strongest hypoxia response”, and intriguingly that NE were an exception in that they “appeared to exhibit the same oxygenation levels regardless of their distance to tracheoles”. 

We have modified the sentence of the trachea elaboration in the OL into “modest ramification” as suggested. We have also mentioned that hypoxia responses in two neuroepithelia appear to stay constant regardless of their distance to the trachea. We also added that among neural cells, NBs express the highest levels of hypoxia responses. 

8. Integration of extrinsic signals with the neuroblast intrinsic proliferation program 
In Figure 5, depiction of oxygen levels as regulating neuroblast reactivation should be removed as that has not been demonstrated. 

We have removed oxygen from figure 4. 

For a complete account of neural progenitor behaviours known to be regulated by tTFs, 
Box 2 should indicate that the temporal state of neuroblasts also defines whether tumour suppressor mutations result in malign neoplasia versus benign hyperplasia (Narbonne-Reveau et al., 2016; Shaw et al., 2018) and that, reciprocally, tumour suppressor mutations or oncogene expression arrest temporal progression (Zacharioudaki et al., 2015; Narbonne-Reveau et al., 2016). 

We have incorporated the information about temporal patterning in NB tumours in Box 1 (CNS temporal patterning in Drosophila) as suggested. 

8.2. Hormonal signalling, temporal patterning, and metabolism regulate neuroblast terminal differentiation 
I believe the two paragraphs on this section, both of which mention Ecdysone signalling and OxPhos, could be written in a more integrated manner. A new paragraph comes as a
surprise since OxPhos and catabolism had already been referred to in this context, whereas readers might expect a new concept. 

We separated this part into two paragraphs to differentiate the effects of ecdysone signalling upon two different peaks of ecdysone: one at the CW and one at the larval-pupal transition. To avoid any confusion, we have modified paragraph 2 and 3 in section 4 such that: 
Paragraph 2: ecdysone signalling at the CW, with emphasize on its effect on the Imp/Syp expression shift. 
Paragraph 3: ecdysone signalling at the larval-pupal transition, with emphasize on its effect on the metabolic reprogramming in neuroblasts. 

8.3. Ecdysteroids and temporal patterning in the generation of neuroblasts in the OL 
It could be interesting to refer here to Zacharioudaki et al., 2015, who found that hyperactivation of Notch signalling can arrest temporal progression in type I and II neuroblasts. Although the OL presents a distinct cellular context, this raises the hypothesis of mechanistic integration between Notch signalling levels, proneural wave propagation and 
temporal transitions in the OL. 

This is a very interesting hypothesis! We have added a part in section 5.1 (paragraph 3) about this possibility of mechanistic integration between Ecdysone and temporal patterning through the regulation of Notch signalling, to modulate proneural wave propagation. 

Conclusions and perspectives 
2. Integrations of intrinsic and extrinsic factors in the regulations of CNS tumour growth 
Worth mentioning here also that BBB disruptions are found in the context of neural tumours. 

We have added as sentence that tumour formation in the CNS can lead to the disruption of the BBB integrity by downregulations of transporters and junctional proteins. 

Regarding the final sentence of this section, note that some of what has been reviewed explains some aspects of the “bypassing” since retention of early temporal factors in a 
fraction of tumour NB-like cells will stabilise Myc and preclude eliciting Mediator. 

Although neuroblast-like cells were shown to have Imp expression persisted beyond the normal developmental timepoint (Genovese et al., 2019), Myc expression has not been reported in this context. We agree that Imp can stabilise myc mRNA in neuroblasts, however, this was shown during neural development (Samuels et al., 2020). Thus, it is still uncertain that Myc is upregulated in Imp+ neuroblast tumours. The same applies to the regulation of Mediator by Imp (Yang et al., 2017). Nevertheless, regulation of Myc and Mediator by the retention of Imp remains possible explanation for the “bypassing” mechanism of tumour neuroblasts (we have added this discussion in the Summary and Perspectives). 


LANGUAGE AND FORMALITIES 
Abstract 
Line 11: would be better as “progenitors that in turn generate differentiated neurons” * 
Lines 21 and 27: remove “the” 
Introduction 
Line 26: refers to the case in mammals so that might be worth making explicit; 
Furthermore, in that case, do they all first divide symmetrically? 
Line 35: “neuronal” should be “neural” to encompass glial cell types 
Line 37: missing comma after “NPs” (changes the meaning slightly) 
Line 52: worth adding “neurogenesis in Drosophila.” at the end of ongoing sentence 
1. NSCs in Drosophila 
Figure 1C: looks like there are NBs in the neuropil; furthermore, note that the acronym “NB” 
has not been introduced 
Page 3 Line 43: best to say that the GMC divides, often asymmetrically, to generate two 
differentiated neurons or glial cells (similar to *) 
Page 3 Line 49: “upon the induction of dietary nutrients” is unclear 
Page 4 Line 4: Similar to * (generation of differentiated progeny rather than “differentiate”) 
Page 4 Line 8: would be better as “a pair of lateral neuroblasts” (as “lateral neuroblasts 
alone could refer to any number) 
Page 4 Line 15: make clear that INPs also divide asymmetrically 
Page 4 Line 20: again should be “neural” rather than “neuronal” as some glia is produced by 
at least one of the type II lineages 
2. The neuroblast local niche 
Heading: by definition a stem cell niche is “local” so would remove that word, which feels 
redundant 
Page 6 Line 15: I suggest “including various types of neurons and glia, as well as tracheal 
cells” 
Page 6 Line 15: missing comma before “comprised” 
Page 6 Line 44: again should be “neural” to be more encompassing (including neuroblasts, 
neurons and other glia) 
7 
3. The local niche regulates neuroblast quiescence and reactivation alongside neuroblast
cell-intrinsic mechanisms 
Page 14 Line 27 and Figure 3: “secret” should be “secrete” 
Page 11 Line 35: “Because neuroblast exit” more elegant than ”neuroblasts’ exit” 
4. Surface glia support neuroblast proliferation beyond reactivation from quiescence 
Page 13 Line 10: first appearance of the acronym TGFb so it should be spelt out here rather 
than in Line 15 
5. Cortex glia regulate neuroblast proliferation 
Page 14 Line 31: “secret” should be “secrete” 
6. Lipid metabolism of the glial niche regulates neuroblast proliferation 
Page 16 Line 13: “On the contrary” would read better as “Conversely” 
Page 16 Line 27: “have been known” would read better as “have been shown” 
Page 16 Line 32: “essential for the Hh” should be “essential for Hh” as this has not yet been 
referred to 
Page 17 Line 6: “poor” should be “poorly” 
Page 17 Line 7: “Acid (PUFA)” should be “Acids (PUFAs)” as there are multiple 
Page 17 Line 22: again PUFAs – should be plural 
7. The local niche regulate development of the OL medulla 
Typo in heading: should be “regulates” 
Page 18 Line 5: instead of “division”, “development” would be preferable 
Page 18 Line 13: “neuroepithelial” should be “neuroepithelium (NE)” 
Page 18 Line 18: “, which” should be deleted; and “preventing precocious neuroblast 
transition” would be clearer as “preventing their precocious transition into neuroblasts” 
Page 18 Line 34: “investigated how” could be “investigated if and how” 
Page 18 Line 46: “autonomously” would be better as “directly” 
Page 19 Line 11: “express the chloride channel ClC-a” should be “express Chloride Channel A 
(ClC-a)” 
8. Integration of extrinsic signals with the neuroblast intrinsic proliferation program 
8.1 heading: better grammar would be “Nutrient-dependent Hippo and PI3K/Akt signalling 
coordinate neuroblast quiescence/reactivation” 
8 
Page 20, Line 55: “coordinately orchestrated” is redundant as “coordination” and 
“orchestration” convey the same message 
Page 21 Line 10: “On the contrary” would read better as “In contrast” 
Page 21 Line 15: “Ribosomal Protein Kinase” should be “Ribosomal Protein S6 Kinase” 
Page 22 Line 6: I would reverse the order to “During neuroblast terminal differentiation 
(also known as decommissioning)” since “termination” is the nomenclature used up to then 
in the article. 
Page 22: note that some text was not visible in main section and Box 2 
Box 1: comma missing after DILPs, “the growth” would read better as just “growth”, 
“growth transition” and “prepares tissue” should be pluralised (“transitions”, “tissues”) 
Page 23 Line 13: “at the end of neurogenesis” is unnecessary (redundant) 
Page 23 Line 31: “of neuroblasts” is unnecessary (redundant) 
Page 23 Line 55: typo in “energic” which should be “energetic”, although “metabolic” might 
be best 
9.3 Heading: should be 8.3 
Page 24 Line 32 and Page 25 Line 13: “differentiate” might be better if replaced by 
“transition” for consistency with prior sections and to avoid confusion with terminal 
differentiation 
Conclusions and perspectives 
Heading: Given that a subsequent section is entitled “Conclusion” the title of this one could 
be edited to “Summary and perspectives” 
Subheadings numbered herein as 1 and 2 should be avoided given this numbering already 
used for article sections (distinct paragraphs without titles might suffice) 
Page 15 Line 24: instead of “recent” it would do the authors more justice to say “we 
summarised data spanning four decades of findings that show” 
Page 25 Line 29: I suggest deleting “the” as a key parameter that regulates neurogenesis not 
discussed as regulated by extrinsic cues was asymmetric division (understandably, given
limited evidence to this effect but “the” sounds as if those listed constitute all the key 
parameters) 
Page 25 Line 33: would read better as “NSC proliferation with niche and systemic growth” 
References 
Reference callouts in text: preferable in chronological order 
Pages numbers are missing in a few and I notice a spurious “%” sign 

All of these typos have been corrected.

AE review

In my view, the reviewers’ concerns have been addressed effectively. The revised manuscript provides a timely summary of extrinsic cue-regulated Drosophila neurogenesis that should be of general interest. I recommend that it is accepted without further revisions.

Decision letter

23-Mar-2022 

Dear Dr. Cheng, 

It is a pleasure to accept your revised manuscript entitled "Non-autonomous regulation of neurogenesis by extrinsic cues: a Drosophila perspective" in its current form for publication in the Oxford Open Neuroscience.  The comments of the reviewer(s) who reviewed your manuscript are included at the foot of this letter. 

Thank you for your fine contribution.  On behalf of the Editors of the Oxford Open Neuroscience, we look forward to your continued contributions to the Journal. 

Sincerely, 
Dr. Hongyan Wang 
Senior Editor, Oxford Open Neuroscience 
hongyan.wang@duke-nus.edu.sg
